# Reliable Multi-Fractal Characterization of Weighted Complex Networks: Algorithms and Implications

**DOI:** 10.1038/s41598-017-07209-5

**Published:** 2017-08-08

**Authors:** Yuankun Xue, Paul Bogdan

**Affiliations:** Ming Hsieh Department of Electrical Engineering, University of Southern California, 90007 CA, USA

## Abstract

Through an elegant geometrical interpretation, the multi-fractal analysis quantifies the spatial and temporal irregularities of the structural and dynamical formation of complex networks. Despite its effectiveness in unweighted networks, the multi-fractal geometry of weighted complex networks, the role of interaction intensity, the influence of the embedding metric spaces and the design of reliable estimation algorithms remain open challenges. To address these challenges, we present a set of reliable multi-fractal estimation algorithms for quantifying the structural complexity and heterogeneity of weighted complex networks. Our methodology uncovers that (i) the weights of complex networks and their underlying metric spaces play a key role in dictating the existence of multi-fractal scaling and (ii) the multi-fractal scaling can be localized in both space and scales. In addition, this multi-fractal characterization framework enables the construction of a scaling-based similarity metric and the identification of community structure of human brain connectome. The detected communities are accurately aligned with the biological brain connectivity patterns. This characterization framework has no constraint on the target network and can thus be leveraged as a basis for both structural and dynamic analysis of networks in a wide spectrum of applications.

## Introduction

Complex systems consist of heterogeneous agents mutually influenced via interactions of different intensities over multiple spatio-temporal scales. This heterogeneity encompassed in both the participating components and their varying interactions makes complex systems difficult to decipher. To understand and control these complex systems, the network theory provides an effective mathematical modeling framework that enables the encoding of the entities (nodes) of a complex system and their heterogeneous interactions (links) of different strength (weights) into a topological network configuration implicitly embedded in metric spaces, where the distance among nodes is decided both by the structural configuration of the system (topology) and the intrinsic nature of the inter-node couplings (e.g., social affinity, chemical bonds, traffic intensity or neural connectivity strength). In some cases, the properties of the inter-couplings among system components and the corresponding spatial embeddings even play a far more dominant role in regulating the overall system behaviors and dynamics. For instance, the atomic and molecular interactions among a chain of amino acids definitively dictate not only the dynamical spatial conformation of the corresponding protein but also its biological functionality^1, 2^. The disturbance of normal protein interactions can lead to irreversible pathological consequences known as proteopathies like Alzheimer’s, Parkinson’s^3^ and Huntington’s disease^4^. Therefore, the study of structural organization, formation and dynamics of the complex systems can benefit from studying their geometrical properties and discovering new relationships between geometrical characteristics and network problems (e.g., community structure identification).

Learning the geometric principles underlying the organization of complex systems modeled by weighted networks facilitates the identification of their fundamental properties. Some of complex networks have been found to be *Small world* or *Ultra-small world*. Small world network model characterizes a graph of size *N* for which its average path length increases proportionally to the logarithm of the number of nodes $\langle d\rangle  \sim logN$. In contrast, the Ultra-small world networks are characterized by smaller shortest path distances that scale as ${d}_{min} \sim loglogN$. Albert Barabasi and his colleagues found that the Erdos-Renyi random network model can not explain the formation of densely interconnected hubs or clusters in a family of real networks with degree distribution obeying a power-law^5^. In contrast to the Erdos-Renyi random network model that leads to a narrow normal degree distribution, the power-law degree distribution of these networks has such a long tail that we cannot reason about the interconnection density of the network given a randomly chosen sample, hence they are *scale-free*.

The discovery of small-world property led to the belief that complex networks are not invariant under a length-scale transformation according to which an exponential dependence holds between the size of the network and its average path length. However, it is found that a variety of real networks exhibit self-repeating patterns at all length scales by applying a renormalization procedure^6, 7^. This illustrates the concept of *self-similarity*. The coexistence of self-similarity and small-world property in a variety of complex networks is further verified^8^. These two contradictory properties call for further investigation on the appropriate mathematical model of complex networks and their main features. A phase transition phenomenon is found between the local self-similarity and the global small-world property by studying the stability of nodes by renormalization group theory^9^.

The uncovered self-similarity in complex networks connects to the important *fractal* and *multi-fractal* geometry domain where a family of objects are distinguished based on their self-repeating patterns and invariability under scale-length operations. Such objects are known as fractal objects. A mono-fractal object obeys a perfect self-repeating law at all scales. When embedded in Euclidean metric space and tiled by equally sized boxes at different scales, it becomes apparent that an important property of fractals is the power-law dependence between the mass distribution *M*(*r*) (e.g., the number of points in a box) and the scale factor *r*:

1 $$M(r) \sim {r}^{D}$$

In Eq. (1), *D* is the *fractal dimension* and represents a real-valued number in contrast to the embedded space dimension which is always an integer. Fractal dimension is the major tool for describing the fractal geometry and the heterogeneity of irregular geometric objects that the dimension of its embedded space fails to capture. For instance, in Euclidean geometry, a straight line and a crooked line share the same geometrical dimension but have very distinct properties. Multi-fractals could be seen as an extension to fractals with increased complexity. They are invariant by translation although a distortion factor *q* needs to be considered to distinguish the details of different regions of the objects as a consequence of *inhomogenous* mass distribution. Intuitively, multi-fractals are *not* perfect self-repetitions but rich in *localized* variations of detailed geometric configurations. Consequently, a single fractal dimension is *not* sufficient to characterize the irregularity of the geometric shapes as the scaling factor measured across the object could be *different*. As a result, multi-fractal analysis (MFA, see Methods for details) is proposed to capture the localized and heterogenous self-similarity by learning a generalized fractal dimension *D*(*q*) under different distortion factors *q*.

**Figure 1:**
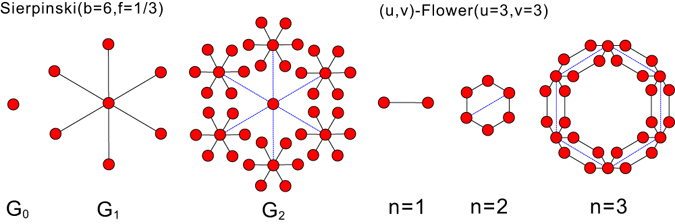
Failure of single (dominant) fractal dimension to capture the heterogeneity in detailed configuration of fractal networks. A comparative example shows. (**a**) Sierpinski fractal network (*s* = 1/3, *b* = 6) and (**b**) (u, v)-flower fractal network (*u* = 3, *v* = 3) share the same fractal dimension (1.631) yet having distinct topological structure.

MFA has been applied to investigate the underlying geometrical principles in a wide spectrum of applications including signal processing^10–13^, imaging processing^14–16^, genomics^17, 18^, geophysics^19, 20^, turbulence analysis^10, 21, 22^, network traffic modeling^23^ and financial analysis^24–27^. Irrespective of the effectiveness of MFA in various domains, its application to study the self-similarity of complex networks is *not* straightforward as the Euclidean metric is not well defined in a topological object like the complex network. The box-covering method was introduced^28^ to calculate the fractal dimension of unweighted complex networks and the authors proved its reducibility to the well-known graph coloring problem, which is NP-hard. However, a single fractal dimension is not a sufficient characterization of self-similarities embedded in the complex network. For instance, how can we distinguish fractal networks that share exactly the same fractal dimension but look entirely different? Fig. 1 shows two fractal networks, namely, *Sierpinski fractal networks and* (*u*, *v*)*-flower*. Both networks have the exact same fractal dimension of *ln*(6)/*ln*(3) ≈ 1.631 but show distinct structural properties. Apparently, relying on mono-fractal analysis does not allow us to distinguish between these two networks. Another relevant question is how link weights affect the fractality/multi-fractality of the weighted complex network. Link weights play an important role in governing the dimension of the network as there exists a mapping from a weighted network to a network spatially embedded where weights translate to the length of links that affects its dimension^29, 30^. To address this problem, an alternative box-covering method (BCANw) was proposed for the numerical determination of the fractal dimension of weighted complex networks^31^. Its application has also been extended to study the inhomogeneity of weighted real-world networks through multi-fractal analysis. Following the same line, a similar study of multi-fractality embedded in weighted networks using the modified sandbox method (SBw) is reported^32^. However, neither of the two methods considers the impact imposed by the distribution of the link weights. Both algorithms are prone to *intrinsic* estimation bias as a consequence of i) ignoring the skewness of the link weight distribution and ii) the implicit assumption that a global fractal/multi-fractal scaling holds at all scales of the network. Moreover, there is no theoretical foundation to support the design and evaluation of both algorithms in order to analyze the factors that adversely impact their numerical accuracy. All these disadvantages leave room for an erroneous characterization of both the structural and dynamical features of the weighted complex networks.

To overcome these issues, we first analytically study the multi-fractal structure of the Sierpinski fractal network family to set up the theoretical ground for evaluation and comparative analysis of our proposed algorithms (See Supplementary Material Section 1). We find that the multi-fractality identified by SBw can be just the side effect of the limited size of the network considered. The analytical discussion of multi-fractality in Sierpinksi family $\mathcal{S}$ provides the theoretical basis on which not only we can quantitatively reason about the existence of multi-fractality/fractality from an asymptotic perspective that numerical approaches will surely fail to offer, but also we can shed some light on the design of numerical algorithms for reliable estimation of multi-fractal spectrum of the complex networks.

To motivate the design of a reliable algorithm which eliminates the disadvantages of BCANw and SBw, we analyze the source of the estimation bias of both algorithms through a set of numerical experiments. We show a compatible growth rule is required to remove the bias, given weighted complex networks of finite resolution. The detailed quantitative error analysis to investigate the source of the intrinsic estimation bias of both algorithms can be found in our Supplementary Material Section 2.

Based on both our theoretical findings and numerical experimental results, we propose the *finite box-covering algorithm for weighted network (FBCw)* and the *finite sandbox algorithm for weighted network (FSBw)* with improved performance. We compare the accuracy of the estimates obtained by FBCw and FSBw with our analytical results of Sierpinski fractal network as well as with those obtained by BCANw and SBw. The comparison shows that the proposed algorithms are not only able to give reliable numerical estimates of fractality with insensitivity to the distribution of link weights, but also are capable of detecting the fractal scaling dependence when it holds within a finite range of scales (i.e., scale-localized).

More importantly, we apply the proposed algorithms to learn the multi-fractal structure of a set of real world weighted networks. We show the link weights play a definitive role in governing the existence of fractality in the network. The investigated weighted networks exhibit a phase transition from self-similar networks to small-world networks when converted to binary networks. Furthermore, we demonstrate that the fractal and multi-fractal scaling behaviors can be *spatially localized* and co-exist in the same network. Learning from our observations on the locality of the scaling behavior of real world weighted networks, we finally propose a network characterization framework based on the localized scaling feature space learned by the construction of scaling feature vectors for each node in the network. The proposed characterization is general and not limited to complex networks that are fractal or multi-fractal. It can be easily interfaced with subsequent analytical tools (e.g., machine learning algorithms) to unveil the intrinsic properties of the weighted complex networks. To illustrate the benefits of our methodology, we apply our algorithms to the network community detection in the human brain connectome. The identified communities are consistent with our biological knowledge.

The following discussion is organized in three parts. In the first part, we present the estimation error of previous numerical algorithms. In the second part of discussion, we compare the performance of BCANw, SBw with the proposed FBCw and FSBw. Finally, we present the multi-fractal analysis on a set of weighted real world complex network and propose a localized scaling based approach for the characterization of the weighted complex networks. We provide an illustrative application example in network community detection to show its effectiveness.

## Results

### Analysis of Finite Resolution and Link Weight Distribution

#### Estimation error analysis and stairway effect

The link weights distribution of complex networks largely depend on the growth rule and weights allocation process. For instance, the distribution of link weights of Sierpinski family is shaped by the scaling factor *s* and growth rule *b*. Interestingly, for small scaling factor *s*, we prove *G*_*k*_ approaches a monofractal that has no explicit dependence on weights distribution (See the proof in Supplementary Material Section 1). Yet this is valid only for a complex network that has *infinite* resolution in the sense that box/sandbox can grow by infinitely small steps (but not continuously) in a network of unbounded range of scales. In most of cases, this does not hold for complex networks and perfect fractals of limited size (e.g., Sierpinski network of limited size). Therefore, when it comes to numerical calculation of the limit in Eqs (13) and (17) using linear regression which is shared by both box-covering and sandbox methods, we are able to show that the box/sandbox should grow in a regulated way that is ***compatible*** with link weights distribution such that the ***stairway*** effect is minimized.

**Figure 2:**
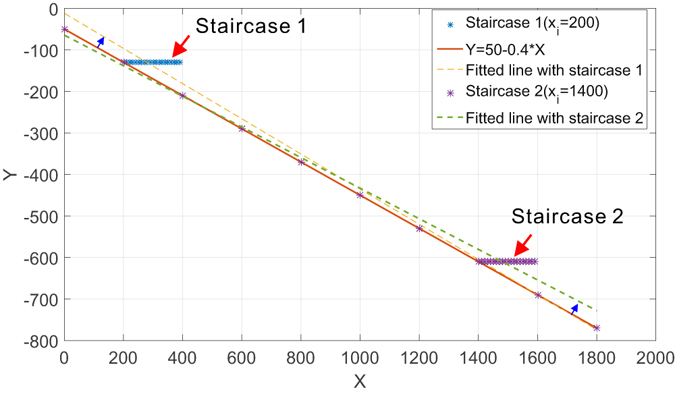
A case study of stairway effect. A linear relation *y* = 50−0.4*x* is observed on a set of input *x*. Two sets of unchanging *y* observations are manually inserted between two actual measurement to create “stairs”. The introduction of such stairs biases the linear regression and causes estimation errors.

The stairway effect is an immediate consequence of applying box-covering or sandbox methods to weighted complex network of finite resolution to estimate the generalized fractal dimensions using linear regression. For a linear regression that minimizes least square error (LSE) ∑ (*y*_*i*_ − *x*_*i*_ *θ*)^2^, stairway effect can be stated as stagnant changes in *y*_*i*_ irrespective of variation of *x*_*i*_ up to a certain range. We show a simple example in Fig. 2 where the output and input observations are made from a linear relation *y* = 50 − 0.4*x*. The solid line shows the perfect fitting when no staircase is introduced. The two dashed lines correspond to the case where a set of unchanging observations are inserted (i.e., fake observations) between two actual *y* observations at two different locations (as denoted by *x*_*i*_), creating two pieces of “staircases” in the plot. As a consequence of minimization of LSE, the fitted lines in presence of staircases deviate from the perfect fitting differently based on the position of the staircases in the plot. We can show the estimation error is proportional to the width of the staircase and the number of fake observations inserted. We have presented the detailed error analysis regarding the stairway effect in our Supplementary Material Section 2.

#### Finite resolution and compatible growth rule

The above mentioned insertion of fake observations can be understood as an interpolation or oversampling process when a system with *finite resolution* is measured. For instance in a linear system, except for the case where input and output are decoupled in the system of interest, a staircase will be created in the measurement if the sampling rate is *not compatible* with the changing rate of the output, e.g., sampling rate is much greater than how the system actually changes its state. The changing rate of the system in this example intrinsically determines its “resolution”.

Similarly, a fundamental difficulty in extending the use of box-covering or sandbox method for determination of multi-fractality in complex network lies in its finite resolution. For a geometric multi-fractal/fractal object embedded in Euclidean space, the probability measure *μ* is well-defined on a continuous interval (0, *L*] where *L* is the length of the object. Alternatively stated, it is possible to grow box size continuously and retrieve the estimates from a linear regression that considers all obtained measurements. However, the distance metric of a complex network is discrete and probability measure *μ* is not defined on a continuously spanning *l* horizon, i.e., for any *l*_*i*_ and *l*_*i*+1_ ∈ (0, *L*], there exist an infinite subset ${L}_{i}=\{{l}_{{i}_{k}}|{l}_{{i}_{k}}\in ({l}_{i},{l}_{i+1})\}$ on which the probability measure *μ* is a constant. In other words, we can not distinguish between any element in this subset based on their associated probability measure, hence the *resolution* is *finite*.

As a consequence, we observe staircases that correspond to the measurements on these subsets *L*_*i*_ if we directly apply box-covering or sandbox method with box size (or equivalently the radius of a sandbox, we use only the term “box size” for short) growing continuously. The presence of staircases, as we discussed earlier, introduces bias into the estimates of generalized fractal dimension. Obviously, we can minimize the stairway effect if we can aptly choose a growth strategy to scale the box size *l* such that no element in ∪*L*_*i*_ is chosen as the size of a box. We call such strategy, if exists, as *compatible growth rule*.

For unweighted networks, a compatible growth rule can be easily found by increasing the box size in a discrete way, i.e., adding one each time. This is feasible because even though the resolution of an unweighted network is limited yet it is *homogenous* across the network, i.e., distance between any directly connected pair of nodes is identical. However, such property does not hold for a weighted complex network due to the *distribution of link weights*. Searching for a compatible growth rule for box size scaling in a weighted network is much more difficult and sometimes impossible. To give some intuition, let us look at sandbox method. Formally, for a weighted network *G* = (*V*, *E*), let us assume *v*_*i*_ ∈ *V* as the center of sandbox and *v*_*j*_ ∈ *V* as a random node. Denote *d*_*i*,*j*_ as the shortest path length between *v*_*i*_ and *v*_*j*_. The shortest path distribution ${F}_{{d}_{i,j}}(l)=P\{{d}_{i,j}\le l\}$ has a discrete support set ${L}_{i}=\{{l}_{k}|{F}_{{d}_{i,j}}({l}_{k})\ne {F}_{{d}_{i,j}}({l}_{k^{\prime} }),\forall k\ne k^{\prime} \}$ defined by *G*. A compatible growth rule of *v*_*i*_ on *G* is thus a strictly ordered set ${L}_{ < ,{v}_{i}}=\{{l}_{1},{l}_{2},\ldots ,{l}_{n}\}$ where *l*_*k*+1_ > *l*_*k*_. Therefore, the compatible sandbox growth rule *L*(*G*) on *G* is defined by $L(G)={\cap }_{i}{L}_{ < ,{v}_{i}}$ where *v*_*i*_ is the center of the sandbox. It should be noted that it is always possible to find a compatible growth rule for a sandbox centered at a fixed point by growing its size by its *unique* distance to all other nodes. However, there is no guarantee that this growth rule is compatible for another sandbox centered differently. A compatible growth rule requires that the support of path length distribution is shared among all choices of sandbox centers. Apparently, if the choice of sandbox in Eq. (17) (see FBCw and FSBw description in Methods section) is randomized over all the nodes in an unweighted graph *G*, *L*(*G*) = {1, 2, 3, …, *d*_*min*_} where *d*_*min*_ is the shortest path length of the longest distance between any pair of nodes of *G*. However, it can be practically difficult to find in a weighted network of *rich heterogeneity* such *L*(*G*) that i) is shared by a sufficiently large subset of *V* to be mathematically consistent with the estimate obtained by the box-covering method and ii) provides large set of samples of *l* to numerically calculate the limit in Eq. (17).

Such heterogeneity connects tightly to the skewness of the underlying *link weight distribution* of *G* that governs not only the existence of *L*(*G*) but also the design of a reliable algorithm to determine the multi-fractality of *G* when *L*(*G*) does not exist. For instance, the unweighted network corresponds to the case where the link distribution is a symmetric delta function, hence the existence of multi-fractality is solely determined by the topological properties of the network. When the link weights are uniformly distributed, the topology of network again determines the multi-fractality of the network. Since the weights are uniformly distributed, *L*(*G*) = {*E*[*w*], 2*E*[*w*], …, *d*_*min*_ *E*[*w*]} is a statistically compatible growth rule. In other cases especially when the distribution is *highly skewed*, a poorly designed algorithm without awareness of link distribution leads to significant estimation errors in Eqs (13) and (17), rendering the basis of multi-fractal analysis questionable. This estimation error can be well-explained by our analytical findings that the incompatible growth rule with presence of a skewed link weight distribution will lead to the stagnant observations in spite of the growing box size of *l*, i.e., the staircase. Wider staircases will produce *underestimated* slope, hence biased estimates of multi-fractality.

Both BCANw and SBw that are previously proposed for numerical determination of multi-fractality in weighted networks fall into this category as they both rely on an incompatible growth rule and do not consider the skewness of link distribution. We show in the following discussion their disadvantages compared to our proposed algorithms. To make fair comparison under the same experimental setting with priorly known ground truth about multi-fractality of the study object, we choose Sierpinski fractal network family (see Supplementary Material Section 1 for details) as our target network. We conduct comparative error analysis across all four algorithms when applied to estimate the dominant fractal dimension of the target networks.

### Comparative Analysis of Estimation Methods

#### Intrinsic estimation bias of BCANw and SBw

**Figure 3:**
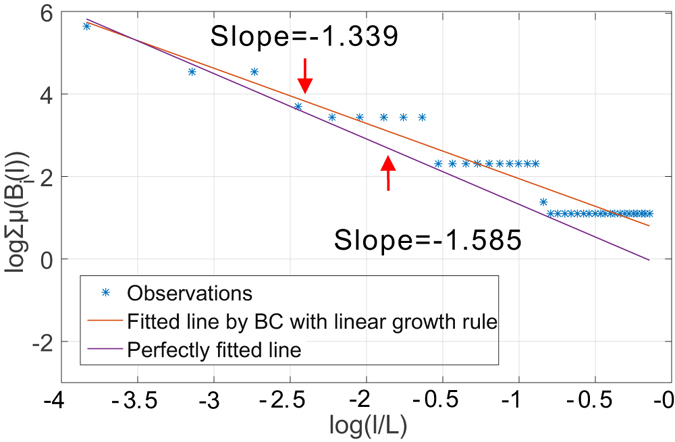
Observation of staircase effect in determination of dominant fractal dimension of *G*_4_ of Sierpinski fractal network family using box-covering method with an incompatible linear growth rule.

**Figure 4:**
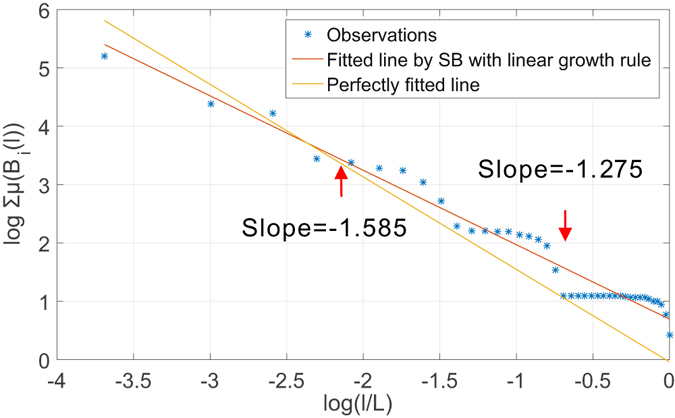
Observation of staircase effect in determination of dominant fractal dimension of *G*_4_ of Sierpinski fractal network family using sandbox method with an incompatible linear growth rule.

To corroborate our argument, we first present two numerical experiments where a simple yet incompatible growth rule is applied to both box-covering and sandbox methods when used for determination of the dominant fractal dimension of Sierpinski network ${G}_{5}\in \mathcal{S}$ with *b* = 3, *s* = 1/2 (see Supplementary Material Section 1 for detailed construction of *G*_5_). The incompatible growth rule increases the size of the box linearly by accumulating a fixed step length. We show in Fig. 3 the estimated fractal dimension using a box-covering method with this growth rule. The theoretical fractal dimension is *ln*(*b*)/*ln*(*s*) ≈ 1.585. As predicted, the staircases are present throughout the scales of *l* considered. To show its impact on the estimates, we plot the fitted line given by the linear regression on collected measurements and a reference line with the theoretical slope. As one notices in the figure, the staircases drive the estimates to deviate from the theoretical slope. We can make similar observations in Fig. 4 that shows the estimated dominant fractal dimension of the *G*_5_ using sandbox method following the same growth rule. The staircases correspond to incompatible choice of box size *l* from the set ∪ *L*_*i*_ for which the probability measure is not defined. As a result, the measure *μ*(*B*(*l*)) remains stagnant irrespective of changes in *l*, introducing estimation errors when linear regression is performed in Eqs (13) and (17) to determine numerically the multi-fractal spectrum.

**Figure 5:**
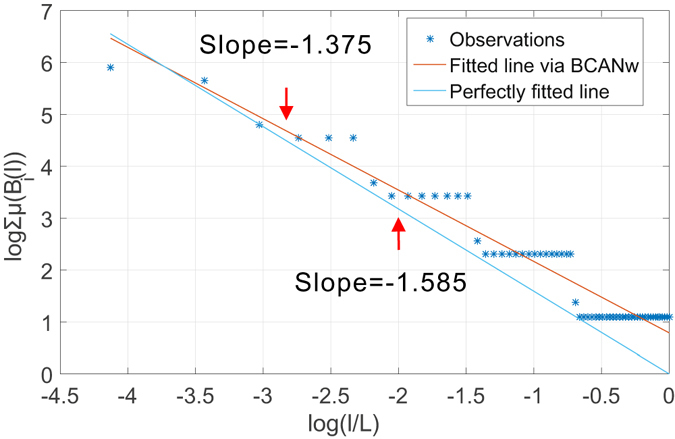
Observation of staircase effect in determination of dominant fractal dimension of *G*_4_ of Sierpinski fractal network family using BCANw.

**Figure 6:**
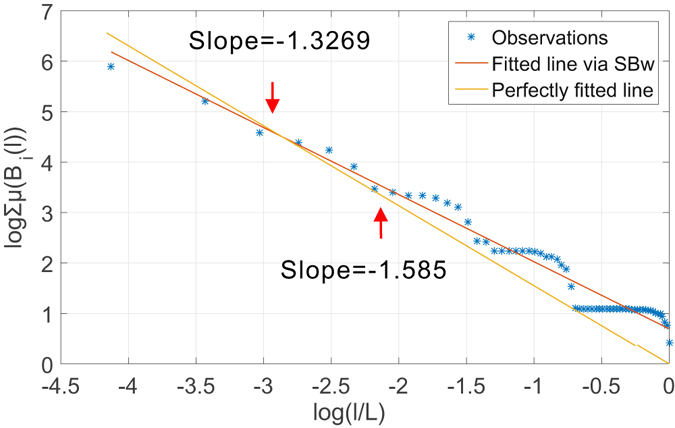
Observation of staircase effect in determination of dominant fractal dimension of *G*_4_ of Sierpinski fractal network family using SBw.

These two simple examples verify our analytical prediction in our analysis of incompatible growth rule when an incompatible growth rule is enforced. In what follows, we show that neither BCANw nor SBw is immune to such incompatible growth rule. Again, we use *G*_5_ as a case study to compare with our simple settings of our first set of experiments. In contrast to a linear growth rule, both BCANw and SBw employ a growth rule *L*_<_  = {*w*_1_, *w*_1_ + *w*_2_, …, ∑_*i*_ *w*_*i*_, ∀ *w*_*i*_ ∈ *W*(*G*)} where *W*(*G*) = {*w*_1_, *w*_2_, *w*_3_, …, *w*_*n*_} is an ordered set of all the weights of *G* such that *w*_*k*_ ≤ *w*_*k*+1_ for all choices of *i*. We apply the BCANw and SBw to estimate the dominant fractal dimension of *G*_5_. To verify the existence of staircase effect when applying BCANw and SBw, we show two case studies in Figs 5 and 6. As one can notice, accumulating the link weights to grow either the box or sandbox is still not compatible with the Sierpinski fractal network *G*_5_. We observe in both experiments that the staircases introduce large bias (1.375 and 1.327 compared to 1.585) in numerical determination of limits in Eqs (13) and (17). The failure to accurately calculate them translates directly to *unreliable estimation* of the *multi-fractal spectrum*.

**Figure 7:**
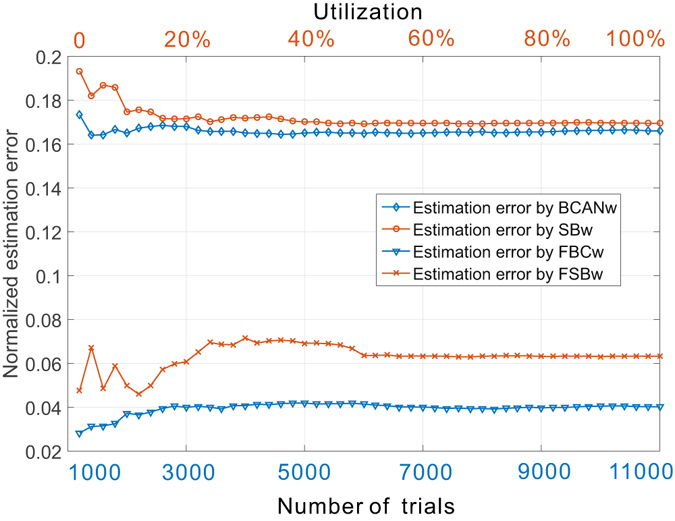
Normalized estimation errors of dominant fractal dimension of *G*_5_ (*b* = 3, *s* = 1/2) under different (i) numbers of the repeated trials for box-covering-based methods (BCANw and FBCw) and (ii) utilization of nodes as sandbox center for sandbox-based methods (SBw and FSBw). Averaging the estimations over an increasing number of box-covering trials or nodes used as sandbox centers brings trivial improvement to the intrinsically biased estimation of BCANw and SBw. The proposed FBCw and FSBw provide better accuracy by addressing the finite resolution and the skewness of the link weight distribution of the weighted complex network.

Moreover, we argue that the estimation errors recognized in Figs 5 and 6 do not come from “infrequent anomalies” of the experiments. Given a fixed size of the network, repeating the experiments using box-covering or averaging the result over an increased set of sandbox centers does not fundamentally compensate the error introduced by BCANw and SBw ignoring finite resolution and link weight distribution of the target network. We performed BCANw with random choice of node coloring order and repeated the experiment by 1000 to 11,000 times with a step length of 200 to obtain the averaged number of boxes to cover the graph to avoid the bias introduced by the deterministic ordering. It should be also noted that the reason we take the average number of boxes comes from the practical consideration. It represents the average performance of the BCANw when it is computationally impossible to repeat the experiments indefinitely to obtain the minimal number of boxes given large-scale networks. We performed SBw with random choice of the center of sandbox from 5% to 100% of nodes in *G*_5_ with a step length of 1.9%. For each step, the results are averaged over 1000 trials. To illustrate the importance of awareness of the finite resolution and link weight distribution on the estimation algorithm, we also performed the proposed FBCw and FSBw with the same settings as BCANw and SBw. The results are plotted in Fig. 7.

We show the estimation errors of the four algorithms normalized against the theoretical dominant fractal dimension. For box-covering based algorithms (BCANw and FBCw, blue lines), we plot the error against different numbers of trials (1000 to 11000). For sandbox-based algorithms (SBw and FSBw, orange lines), the error is plotted against utilization of total number of nodes used as the candidates for the sandbox center. Several key observations can be made for BCANw and SBw: i) As the averaging is performed over an increasing number of trials or sandbox centers, the estimation error is improved slightly. For BCANw, this can be understood as the randomization helps remove the bias of the ordering by which we check the nodes to assign box ID. For SBw, the improvement on estimation error is more significant (from 19.3% to 17%) while it eventually approaches that of BCANw. This is well aligned with Eq. (17) in that the randomized choice of sandbox is the necessary condition for the equivalency of sandbox method to box-covering method. ii) Even though the improvements on estimation accuracy are observed for both BCANw and SBw as the averaging helps remove the random bias, they are trivially small. The randomization is not able to fundamentally compensate for the estimation errors introduced by their incompatible growth rules. Therefore, the staircase effect from the case studies presented in Figs 5 and 6 is the *intrinsic estimation bias* of both algorithms. iii) In contrast, the proposed FBCw and FSBw algorithms consistently outperform the BCANw and SBw by a larger margin. The worst-case normalized estimation error of FBCw is less than 4% and that of FSBw is less than 7%.

The experimental results show that the state-of-the-art BCANw and SBw methods are not immune to errors due to the influence of finite resolution and link weight distribution, hence suffering from the intrinsic estimation bias. It should be noted that these biased estimations can be noticed *only if we know the ground truth* of multi-fractality of the interested network. Such ground truth can hardly be reached if i) we have no access to estimation approaches with optimality guarantee (e.g., optimal box-covering or sandbox methods with compatible growth rule) and/or ii) the underlying mechanism that regulates the growth of the network is unknown or changing over time (e.g., non-deterministic). For weighted fractal networks that extend themselves based on simple rules (e.g., Sierpinski fractal family), our theoretical multi-fractal analysis (see Supplementary Material Section 1) shows that it is possible to develop an optimal approach based on which the ground truth (i.e., the theoretical multi-fractality) can be obtained. However, for most of real-world weighted complex networks there is no such ground truth against which we can compare our estimation of multi-fractality and it is practically very difficult to develop algorithms with optimality guarantees. As a consequence, the bias, which is very likely to exist when BCANw and SBw is used, can hardly be identified hence leading us to unreliable conclusions about the target networks and the urgent need for reliable numerical estimation approaches. As a case study, Fig. 7 already showed the advantage of the proposed algorithms in estimating the fractal dimension of *G*_5_. In what follows, we present a more comprehensive set of comparative analysis on the proposed FBCw and FSBw against BCANw and SBw.

#### FBCw and FSBw for weighted complex network of finite resolution

To further validate the proposed FBCw and FSBw based on the known ground truth about the fractality of the target network, we consider the Sierpinski fractal family with ranged variations in the size of the graph and the skewness of link weight distribution. Formally, the skewness of the distribution is a measure of the *asymmetry* of the probability distribution of a real-valued random variable about its mean. We introduce the Pearson’s moment coefficient of skewness *γ* as the measure of the asymmetry in link weight distributions as follows:

2 $$\gamma =\frac{E[(X-\mu {)}^{3}]}{E{[(X-\mu {)}^{2}]}^{\mathrm{3/2}}}=\frac{{\kappa }_{3}}{{\kappa }_{2}^{\mathrm{3/2}}}$$

*κ*_*t*_ are the *t*-th cumulants. The probability distribution with positive skewness usually has a longer right tail or the mass of the distribution is concentrated on the left of the distribution.

**Figure 8:**
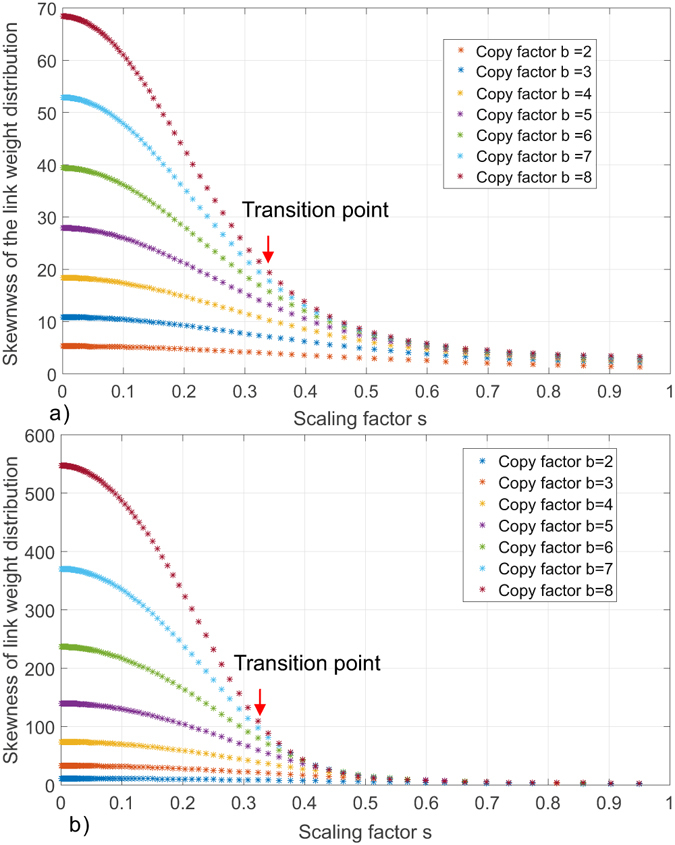
Skewness of link weight distribution of Sierpinski fractal network. (**a**) The skewness of link weight distribution of *G*_5_ as function of scaling factor *s* and copy factor *b* = 2, 3, 4, 5, 6, 7, 8. (**b**) The skewness of link weight distribution of *G*_7_ as function of scaling factor *s* and copy factor *b* = 2, 3, 4, 5, 6, 7, 8.

We extensively measured the skewness of link weight distribution of the *G*_5_ and *G*_8_ under different copy factor (*b* = 2 to 8) and scaling factor *s* ranging from 0.95 to 4.5 × 10^−4^. We report the results in Fig. 8. We can observe that: i) the skewness of the link weight distribution of Sierpinski fractal network increases as the scaling factor decreases and the size of the network grows. Figure 8(a) shows the skewness of link weight distribution of *G*_5_. The smaller scaling factor leads to a less asymmetric distribution. The large copy factor further amplifies this skewness by placing more mass on the left side of distribution (i.e., links with small weights). Similarly, Fig. 8(b) shows a significantly increased skewness in large networks compared to Fig. 8(a). (ii) The skewness of link weight distribution does not increase linearly as the scaling factor decreases. A *transition phenomenon* can be observed as the scaling factor decreases. The skewness grows much slower at large *s* and seems insensitive to the change of copy factors. This observation suggests some potential underlying *phase transition* of the Sierpinski fractal networks as scaling factor decreases. We show a case study in Supplementary Material Section 3 by associating the scaling factor to the free energy of a multi-fractal and study its first-order discontinuity. We observe that there exists a critical scaling factor *s* that describes a transition from a mono-fractal phase to a multi-fractal phase (when considering networks of limited size) of Sierpinski fractal network. (iii) For the same copy factor, the skewness of the link weight distribution tends to converge as the scaling factor decreases. The copy factor *b* dominantly influences the skewness of the link distribution when the scaling factor is small.

**Figure 9:**
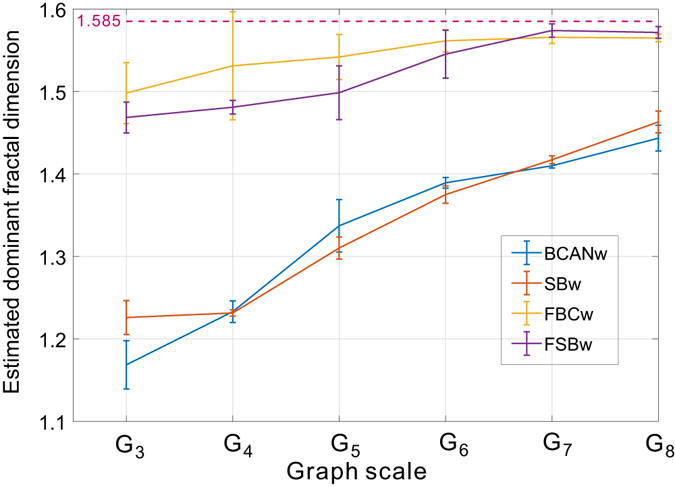
Estimated dominant fractal dimension of Sierpinski fractal network family (*G*_3_ to *G*_8_ with *b* = 3 and *s* = 1/2) using BCANw, SBw, FBCw and FSBw. As predicted by Eqs (13) and (17), the estimation accuracy is improved as the numerical calculation of the limit by the linear regression is performed over a growing set of observations. However, the increased skewness of link weight distribution prevents BCANw and SBw from approaching the theoretical value as quickly as the proposed FBCw and FSBw do.

Figure 8 also shows that the skewness of the link distribution of Sierpinski fractal network is affected by the size of the graph, copy factor *b* and scaling factor *s*. In order to understand how this skewness has impact on the numerical determination of the multi-fractality and compare our proposed FBCw and FSBw with BCANw and SBw, we present two comparative experiments. In the first experiment, we consider a set of Sierpinski family members ranging from *G*_3_ (39 nodes) to *G*_8_ (9840 nodes) given the fixed copy factor *b* = 3 and scaling factor *s* = 1/2. The estimated fractal dimensions are reported in Fig. 9 for BCANw (blue line), SBw (orange line), FBCw (yellow line) and FSBw (magenta line), respectively. For comparison purpose, we also show the theoretical dominant fractal dimension of the target networks with the dashed line. Based on the results in Fig. 9, we can make the following observations:

(i) The proposed FBCw and FSBw are *less sensitive* to the size of target graph compared to BCANw and SBw. The normalized estimation errors of FBCw and FSBw performed on *G*_3_ with only 39 nodes are 5.24% (averaged estimated fractal dimension = 1.50) and 6.56% (averaged estimated fractal dimension = 1.48), respectively. In contrast, the estimation errors of BCANw and SBw are 25.6% (1.18) and 22.4% (1.23), respectively. This property of the proposed FBCw and FSBw is very important in practice when used as the basis of multi-fractality analysis on real networks for which we have neither ground truth to reason about the estimation error nor scaling methods to improve the accuracy. It is critical to have algorithms that have no strict constraint on the target network and deliver reliable estimates in various settings.
(ii) As the graph size grows, the accuracy of all four algorithms is improved as a consequence of more observations obtained to perform the linear regression. This is aligned with Eqs (13) and (17) in that the numerical calculation of the limit in both equations is asymptotically equal to the theoretical value given the linear regression performed on a network member *G*_∞_ of the Sierpinski fractal network with unbounded size. However, it should be also noted that the BCANw and SBw still suffer from significant estimation errors compared to the theoretical value in spite of a large-scale target network (e.g., *G*_8_). Combined with Fig. 8, one primary influencing factor is the increased skewness of link weight distribution of a larger network that will worsen the performance of box-covering and sandbox algorithms with no compatible growth rule. In contrast, FBCw and FSBw quickly converge to the theoretical value with very small errors.

**Figure 10:**
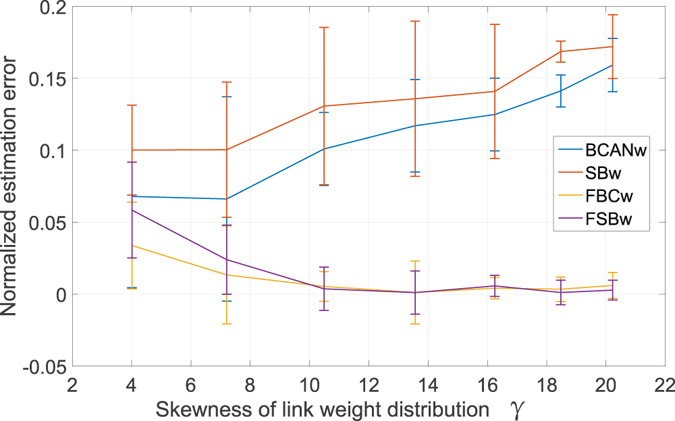
Normalized estimation error of BCANw, SBw, FBCw and FSBw under different skewness *γ* of link weight distribution by changing the copy factor *b* of *G*_5_ from 2 to 8. (i) The performance of BCANw and SBw degrade as the *γ* grows. (ii) BCANw and SBw tend to underestimate the dominant fractal dimension which is aligned with our theoretical prediction in analysis of the staircase effect. (iii) The proposed FBCw and FSBw tends to be insensitive to the change of *γ* and benefit from the increased size of the target network.

To further corroborate our discussion on the adverse impact of skewed link weight distribution on the accuracy of BCANw and SBw, we present the second set of experiments. The experimental setup is motivated by the observation we made in Fig. 8 that the skewness of link weight distribution is dominantly affected by the copy factor *b*. Therefore, we choose a member network from the Sierpinski fractal network family as the seed network. We adopt different values for the copy factor *b* with a fixed scaling factor *s* = 1/3 to generate an array of fractal networks. Then BCANw, SBw and the proposed FBCw and FSBw are employed to estimate the dominant fractal dimensions of all generated networks. Due to the constraint of the computing power, we choose *G*_5_ as seed network and the copy factor ranges from 2 (62 nodes) to 8 (37448 nodes). We report in Fig. 10 the normalized estimation error against the corresponding skewness of the distribution *γ* for BCANw (blue line), SBw (orange line), FBCw (yellow line) and FSBw (magenta line), respectively. Figure 10 can be interpreted as follows. First, by increasing the copy factor one can notice a further skewed link weight distribution. As a result, the estimation accuracy of BCANw and SBw degrades accordingly. The normalized estimation error of BCANw grows from 6.79% (*γ* = 4.0103, *b* = 2) to 15.92% (*γ* = 20.2235, *b* = 8). Similarly, the degradation of estimation accuracy of SBw is worse than BCANw. The error increases from 10.01% to 17.2%. Second, the performance of FBCw and FSBw is not adversely impacted by the increased *γ*. Interestingly, the accuracy is improved as the copy factor increases. This improvement is discussed in our first set of experiments as a result of larger set of observations obtained for more reliable calculation of limit in Eqs (13) and (17).

It is very important to note that both BCANw and SBw *underestimate* the dominant fractal dimension, which is predicted by our analytical findings in estimation errors analysis section. The incompatible growth rule of BCANw and SBw gives rise to the larger set of stagnant observations (i.e., wider staircase) when the skewness is positively higher. A network with higher positive skewness of link weight distribution has more links with smaller weights. SBw grows the sandbox by accumulating the link weights in an ascending order. In presence of highly (and positively) skewed link weight distribution, it might take a large amount of iterations to grow from *l*_*i*_ to *l*_*i*′_ such that the probability measure *μ*(*B*_*i*_(*l*_*i*_)) is not equal to *μ*(*B*_*i*_(*l*_*i*′_)). All the observations generated between *l*_*i*_ and *l*_*i*′_ become stagnant observations or staircases. The more positively skewed the distribution is, the wider the staircases will be and SBw is more likely to underestimate fractal dimension of the network, which is well aligned with our observations in Fig. 10. For the similar reason, even though BCANw grows the box size by accumulating the unique distance in an ascending order, yet we have seen in Fig. 5 that BCANw can not eliminate the staircase effect thus it is prone to underestimate the fractal dimensions as the network becomes more skewed in terms of the link weight distribution.

We have validated the proposed FBCw and FSBw by showing we can obtain fractality estimation of better accuracy over the established BCANw and SBw for the weighted complex networks. In the following discussion, we will employ the proposed FSBw and FBCw for numerical identification of multi-fractality in a set of real-world complex networks.

### Multi-fractal analysis of real networks

#### Vision and objectives of the multi-fractal analysis

Multi-fractality is deeply rooted in the intrinsic heterogeneity of the networks. More specifically, the non-uniformness of the network structure serves as a major source for a spectrum of distinct self-similarities embedded in different regions of the network at a variety of scales. This embedded heterogeneous self-similarities can be identified through the multi-fractal analysis. Intuitively, multi-fractal analysis can be understood as a microscope with an array of distorting filters that pick up a set of distinct scaling behaviors from corresponding parts of the network by changing the distortion factor *q*. A perfect geometric or topological fractal (e.g., fractal networks) shares the same scaling behavior (i.e., the dominant fractal dimension) that is immune to the changes of *q*, suggesting a consistent self-similarity across the network. Such geometric or topological consistency in self-similarity is usually a result of a common underlying growing rule throughout the scales of the network considered (e.g., Sierpinski fractal network). However, such well-preserved growth rule is rarely found (e.g., non-fractal networks) or inconsistent (e.g., coexistence of small-world and fractal properties with phase transitions) in the real-world networks due to the complicated network formation process. This generation process cradles for the intrinsic heterogeneity in both the structural (e.g., network clusters, communities, hubs) and dynamical (e.g., network control, robustness) aspects of the real-world networks. In the following discussion, we will focus on the structural aspects of a set of weighted real-world networks to answer the following three key research questions:

(i) Whether the multi-fractal scaling behaviors can be observed in the target network and how can they be exploited for betterment of our understanding on the structural properties of real networks?
(ii) What is the contribution of link weight to such scaling behaviors if verified in (i) and how will the change of link weight fundamentally impact the observed multi-fractality?
(iii) How can the identification of the multi-fractality be leveraged to supplement our characterization of the real-world complex networks and provide a novel perspective and a practical probe for unveiling their under-explored structural organization?

To study these questions, we choose two weighted real-world networks. The first weighted network is a scientific collaboration network in astrophysics with 16705 nodes and 111252 edges. Each node represents an author and an edge connects two nodes if they published one or more papers together. The weight between any pair of nodes is determined by,

3 $${w}_{i,j}=\sum _{k}\frac{{\delta }_{i,k}{\delta }_{j,k}}{{n}_{k}}$$

*n*_*k*_ is the number of authors of *k*-th paper. *δ*_*i*,*k*_ = 1 only if author *i* co-authored the *k*-th paper and it is 0 otherwise. The weight quantifies how frequently and closely two authors collaborate. The second weighted network comes from the Budapest Reference Connectome v3.0 which generates the common edges of the connectomes of 1015 vertices, computed from the MRI of the 477 subjects of the Human Connectome Project’s 500-subject release. For each edge *e*_*i*,*j*_, the weight *w*_*i*,*j*_ is based on the electrical connectivity of two nodes and calculated by the number of fibers *n* divided by the average fiber length *l*.

#### Space-localized multi-fractal scaling

To address the three major research questions, we consider three sets of experiments. In the first set of experiments, we study whether the target weighted networks in two different domains show any fractal or multi-fractal scaling behaviors. Towards this goal, we applied the proposed FBCw to both networks in order to learn the scaling dependence as expected by Eq. (13) when the distorting factor *q* is varied within a finite range from −10 to 10 with a step length of 0.1. A key observation on Eq. (13) is that the role of the distorting factor *q* connects primarily to the identification of the non-uniformness of the probability measure *μ*(*B*) defined on the support of the weighted networks. Such non-uniformness of the measure and their distinct scaling dependence over the interested scales *l* arbitrates the existence and properties of the multi-fractality in the target network. If the measure *μ*(*B*) is otherwise uniform at all scales, Eq. (13) will not be affected by the choice of *q*, hence learning only the mono-fractality of the support.

**Figure 11:**
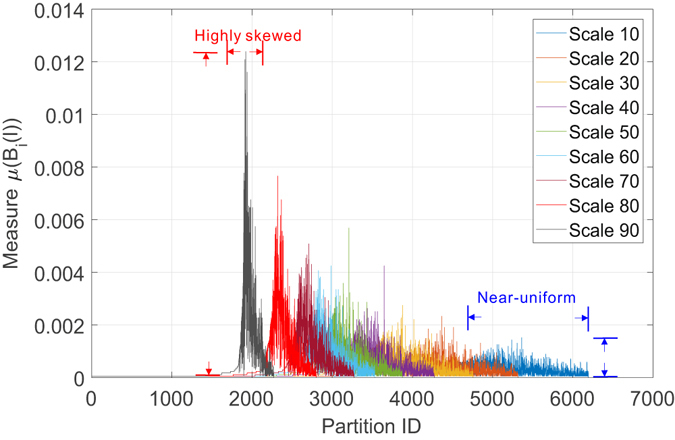
Distribution of probability measure as a function of scale of multi-fractal analysis on collaboration network.

Motivated by such observations, we first look at the weighted collaboration network and report in Fig. 11 the distribution of the measure *μ*(*B*(*l*)) over the partitions (i.e., boxes) of different scales (i.e., size of the box) to give an undistorted overview of the non-uniformness of the measure. Several key observations are due: i) the distribution of the measure *μ*(*B*(*l*)) changes from a near-uniform distribution to a peak shape as the scale increases. Alternatively stated, the probability to find any node in a given box at a specific scale *l* is also a function of the choice of that box. At almost each scale, there exists a partition that contains the dominant number of nodes. This scaling skewness of the measure strongly suggests the structural heterogeneity of the target network and serves as a necessary condition for the emergence of multi-fractality. ii) The rightmost X-axis boundary of the measure distribution marks the minimal number of partitions of scale *l* required to cover the target network. By learning the shrinking law of these boundaries, we can have a straightforward way to verify the existence of fractal scaling behavior. More precisely, we can notice that the rightmost boundaries of the measure do not shrink as quickly as an exponential function but following a power law (which is much slower as indicated in Fig. 11). The corroboration of these two observations demonstrates the existence of multi-fractal behavior in the collaboration network.

**Figure 12:**
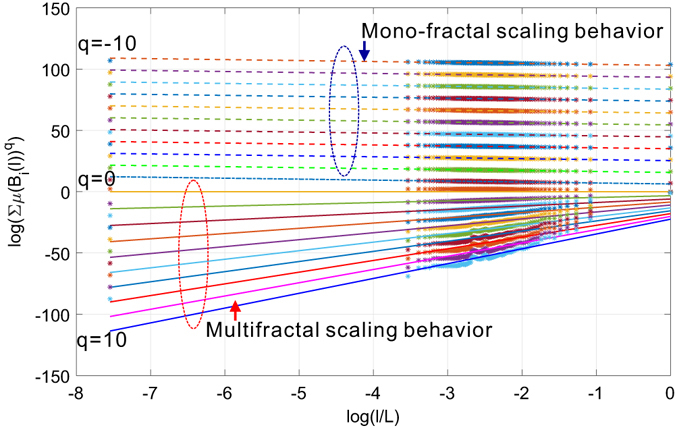
Coexistence of multi-fractal and mono-fractal scaling in the collaboration network.

To further investigate the mulitfractality of the collaboration network, we performed the follow-up experiment to report the scaling dependence between ∑ *μ*(*B*)^*q*^ against the normalized box size *l*/*L* in a *log* − *log* plot under different distorting factors *q*. For ease of visualization and readability, we construct the plots by choosing only the cases when *q* is integer-valued. The results are plotted in Fig. 12. We can observe that the logarithmic distorted accumulative measure *log*(∑ *μ*(*B*)^*q*^) has a linear dependence that is almost *immune* to the changes of negative *q* on the normalized scale *log*(*l*/*L*), suggesting a mono-fractal scaling behavior. However, such linear dependence still holds and is subject to remarkable changes as a function of positive *q*, which is an indicator of the existence of multi-fractality. To understand this, we need to link this observation with Eq. (13). Negative distorting factor *q* places greater weights to the partitions with smaller measures whereas does the opposite when positively valued. In other words, we are able to learn distinct scaling dependence of different regions of the measure distribution, which again correspond to different parts of the target network. In our case when *q* is positively valued, the observed multi-fractal scaling dependence corresponds to the partitions of the collaboration network with dominant probability measures. In contrast, the mono-fractal scaling behavior is strongly related to partitions with small probability measures. The two sets of distinct scaling behaviors not only verify the multi-fractal scaling dependence of target network but also suggest a *co-existence* of multi-fractality and mono-fractality in the same network while belonging to different parts of the network. To understand this, we need to look at how the partition is done to tile the target network with box-covering method. Eq. (13) holds only if the covering is optimal (i.e., with minimal number of boxes, see Definition 5). To achieve this, each box has to be as compact as possible such that it covers the maximal possible number of nodes in a connected component. Such connected components might coincide with regions of the network that are highly clustered such that their scaling follows power-laws characterized by different exponents, hence exhibiting multi-fractal behaviors. In contrast, the non-compact box covers nodes that failed to be reached by nodes in these connected components and demonstrate a shared mono-fractal scaling. In other words, it is the intrinsic variations of the network structure that contribute to the observed co-existence of distinct scaling dependence such that the observed multi-fractal scaling dependence is *space-localized*.

The exact mathematical explanation for the coexistence of mono-fractality and multi-fractality calls for a more sophisticated understanding of the underlying network formation mechanism, which is beyond the scope of this work and remains as a future extension.

#### Scale-localized inconsistent multi-fractality of weighted real networks

**Figure 13:**
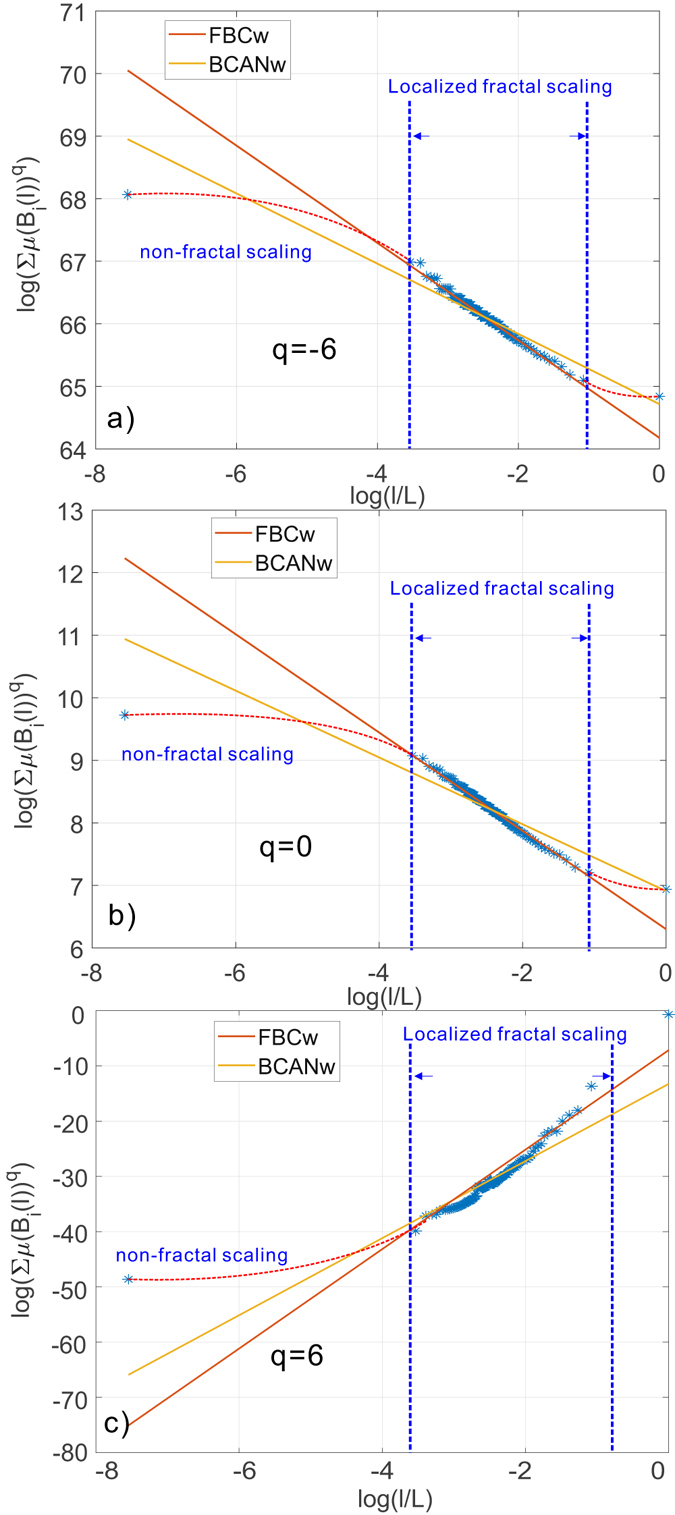
The failure of BCANw to capture the localized fractal scaling of collaboration network over a *finite* range of network scales. In the case of real world networks, the self-similar property does not holds at all scales of networks. There might exist a finite range of scales where fractal scaling behavior dominates. Moreover, this phase transition phenomenon consistently holds under all distorting factor *q*, suggesting a *localized* multi-fractality.

We not only observed the inconsistency of scaling behaviors in different regions of the network, but also we noticed that such scaling is not consistent over the interested scales even when the network exhibits same type of scaling (mono-fractal or multi-fractal) with a fixed *q*. More specifically, we observed the existence of a finite range of scales at which a *localized* scaling behavior holds. To better illustrate this, we have specifically picked cases when *q* = −6, 0, and 6 and reported the scaling dependence in a *log* − *log* plot for each of them. The results are shown in Fig. 13. We use the dashed blue lines to show the range of scales where the fractal scaling appears and the red dashed lines to show outliners. Consequently, we can make the following two observations: (**i**) Fig. 13(a–c) consistently show that the self-similar property does not hold at *all scales* of networks and it might only show up in a finite range of scales. Phase-transition behavior can be observed on boundaries of this range. Moreover, such phase transition phenomenon also holds under a variety of distorting factor *q*. In other words, the multi-fractality of the collaboration network is *scale-localized*. This finding resonates with our claims at the beginning of this section that there is *no* common underlying growth rule for the generation of real networks to produce simple self-repeating structures at all scales of the real networks. As we observed in Figs 12 and 13, the self-similarity is neither spatially consistent across the network nor well-preserved at all scales of the network. (**ii**) In such cases, it is *not* sufficient to have an algorithm that can reliably estimate the scaling dependence *when it exists*. It is also primarily important for the algorithm to *detect* the boundary of scales between which such scaling dependence holds and make a *localized* estimation accordingly. We argue that BCANw ignored such localized fractal scaling by the implicit assumption that *scaling behavior holds at all scales of the complex network*. In contrast, our proposed FBCw is able to locate the phase-transitional scales based on which a reliable estimate is therefore made. To demonstrate this, we performed the BCANw and FBCw on the same network under identical experimental settings. We plotted the fitted linear functions by two algorithms in Fig. 13(a–c). Biased by the implicit assumption that fractal scaling holds at all scales, BCANw tends to average the estimates by considering all the observations, irrespective of their contribution to the fractal scaling behaviors. In comparison, the proposed FBCw detected the locality of multi-fractality and ignored the outliners in the observations not belonging to the range where fractal scaling holds and fit a linear function only to those within it. The difference between the two fitted lines shows the estimation bias of BCANw by assuming a fractal scaling held at all scales.

**Figure 14:**
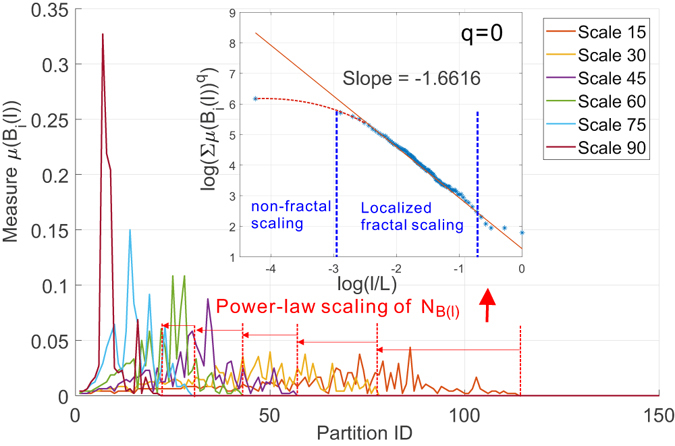
Distribution of probability measure as a function of scale of multi-fractal analysis on Budapest connectome network.

**Figure 15:**
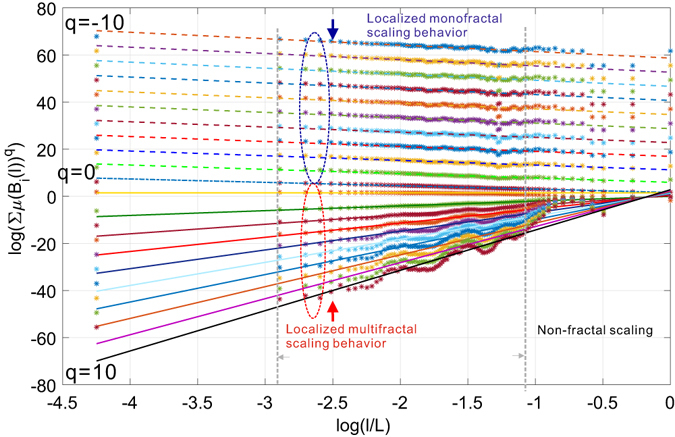
Coexistence of localized multi-fractal and mono-fractal scaling in the Budapest connectome network.

We have repeated the above experiments on the Budapest human connectome network and report the results in Figs 14 and 15. Figure 14 depicts the uniform distribution of probability measure of partitions for the Budapest brain network. Similar to our observations on the collaboration network, the distribution of measure shifts from a near-uniform distribution to a peak shaped non-uniform distribution, suggesting the underlying structural heterogeneity of the brain network, which serves as the major source of multi-fractality. By learning the shrinking behavior of the rightmost boundary of the measure distribution as the size of the box increases, one can observe a power-law dependence which is verified by the subfigure in Fig. 14 where we plot the scaling dependence between the accumulative measure ∑*μ*(*B*)^*q*^ and the normalized scale *l*/*L* in a *log* − *log* scale. Figure 14 is well aligned with our findings in the collaboration network. Budapest brain network also exhibits a *localized* fractal scaling over the ranged delimited by a pair of dashed blue lines, suggesting an inconsistent power-law scaling behavior valid only over a finite range of scales. The fractal organization of brain network is well reported in the related literature. However, few prior efforts have identified the localized fractal scaling given a weighted brain network. To study the multi-fractality of the Budapest brain network, we performed the multi-fractal analysis on it and reported the scaling dependence under different choice of distorting factor *q* in Fig. 15. We can identify the similar co-existence of mono-fractal and multi-fractal scaling as we did in collaboration network. The network regions that correspond to the partition with small measure follow dominantly a near-invariant power-law scaling dependence on the scale of the box. In contrast, the collections of densely connected nodes (e.g., connected components) compactly covered by the box shows a varying power-law dependence as *q* positively changes. However, a major distinction from the collaboration network is the range of scales where such power-law holds. As one can notice, there exists a scale around −1 depicted by the grey dashed line such that the fractal scaling is not respected any more. No linear function could accurately explain the scaling dependence after this scale. This is aligned with our finding in Fig. 14, suggesting a scale-localized fractal scaling behavior that is not globally consistent.

#### Link weight to dictate the mulifractality

**Figure 16:**
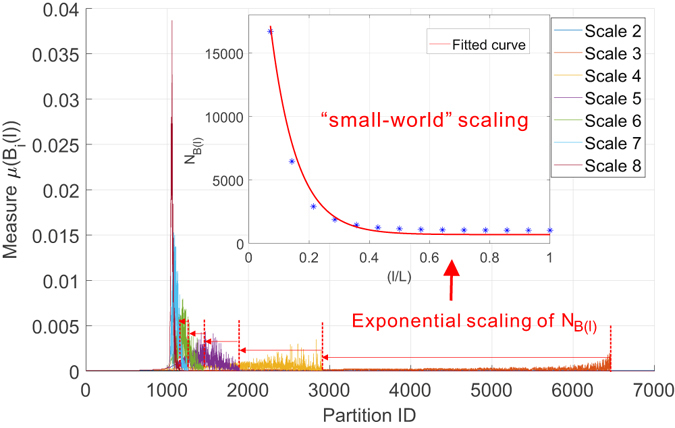
The fundamental impact of link weights on the multi-fractality of network. We keep the exactly same structure of the collaboration network but remove all its weights to transform the network into a binary network. We performed the proposed box-covering method to measure the scaling dependence of number of boxes and the distribution of their associated measure. Figure shows the *loss* of multi-fractality as a result of removal of link weights. Instead, we notice it the scaling dependence is best explained by an exponential law (*a* * *exp*(*bx*), *a* = 3.75 * 10^4^, *b* = −11.55) suggesting the unweighted collaboration network becomes a “small-world” network.

**Figure 17:**
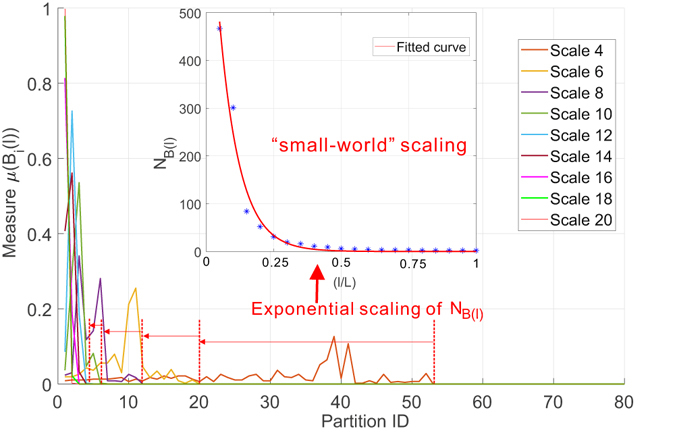
The fundamental impact of link weights on the multi-fractality of Budapest connectome network. Figure shows the similar loss of multi-fractality by removing the weights on the links of Budapest connectome. The scaling behavior is best explained by an exponential law (*a* * *exp*(*bx*), *a* = 934.7, *b* = −0.664) indicating that the common brain connectome is a “small-world” if no weights are considered.

In the second set of experiments, we investigate how the link weights could fundamentally influence the underlying multi-fractality in both networks. Towards this end, we transformed both networks into binary (i.e., unweighted) networks by removing the link weight between any pair of connected nodes. From the geometrical perspective, the link weights on the graph perform a scale transformation to the graph by increasing or decreasing the length of the links when spatially embedded while keeping its topological feature intact. By removing the link weights, we are studying existence of the multi-fractality from a pure topological perspective to understand the role of link weights via comparative analysis. More specifically, we measured the distribution of the probability measure and studied the scaling dependence between minimal number of boxes covering the network and the scale of the box. Figures 16 and 17 summarize the results for collaboration network and Budapest brain network, respectively. Thus, we make the following observations: i) The distribution of probability measure follows a similar changing pattern as the scale of box changes, i.e., from a near-uniform shape to a highly non-uniform shape. This is well aligned with our claims that such non-uniformness is a reflection of structural heterogeneity determined majorly by the topology of the network, which stays intact during our transformation. ii) However, the scaling dependence is fundamentally changed and we observed a total loss of fractal scaling behavior. Instead, the scaling can be well explained by an exponential law, indicating that both the collaboration network and the brain network behave as the well-known *small-world* networks. Compared with their weighted versions, the role of link weight is powerful in dictating the existence of multi-fractality in real networks. Consequently, this finding not only calls for developing new algorithms for estimating reliably the multi-fractal characteristics of weighted complex networks, but also highlights the importance of understanding the structural implications of the identified multi-fractality. This brings us to the following discussion on the third research question of this work.

#### Localized scaling based network characterization and community detection

In the third set of experiments, we study how the multi-fractal analysis framework can be employed to characterize the complexity of networks beyond simply reporting whether they follow a multi-fractal/fractal scaling as many previous works did. We use the multi-scale analysis to quantify the global complexity of the network from a microscopic point of view. Based on the analysis, we proposed a general network characterization framework based on the localized scaling feature space constructed by learning the localized scaling feature vector for each node.

We noticed in the first two sets of experiments that a real world network is *complex* in the sense that there exists no common growth rule that governs the evolution of the network generation process consistently in both the scale and space domain. Similar to our two target examples when certain variation or transformation introduced into the network, the fundamental structural behaviors of the network are subject to remarkable mutations. No single model or characterization is sufficient to fully understand the structural variations and their resulting complexity of the network, hence calling for a set of expressive characterizing strategies that supplement each other to give an unbiased and well-quantified overview of the complex networks. We strongly believe that multi-fractal analysis is a powerful framework to learn the localized scaling behavior and quantify the structural variations of the complex networks.

On one hand, at the microscopic level, the complexity of the network is embedded in the form of different *chemical environments* (i.e., the outer environment surrounding a given node) that each node interacts at *different scales*. More intuitively, the structural variations of the network can be understood as distinct views that a node observes with a lens of variable focal length ranging from the minimal path distance of the network up to its diameter. If all the nodes share the identical viewing experiences with such lens, then the network should have no structural variations like an unweighted lattice which can be fully characterized by its dimension. Otherwise, such microscopic differences in views at a variety of scales, when integrated collectively, translate into the observed structural variations from a global perspective that require multifaceted characterizations.

On the other hand, multi-fractal analysis framework is exactly one of such multi-scale techniques to study and quantify the microscopic proxy of network complexity in terms of structural variations. From the mathematical point of view, Eqs (13) and (17) suggest the structural variations (i.e., structural heterogeneity and link weight distribution) of the networks are the major contributor of the observed scaling behaviors in the complex networks. These variations are distributed in an inhomogeneous way and repeat locally and imperfectly (i.e., space-localized) within a finite range of scales (i.e., scale-localized). Reversely, multi-fractal analysis also provides a way to characterize such structural variation by identifying and quantifying the scaling behaviors (again, not necessarily fractal and/or multi-fractal). More specifically, the proposed SBw method for the weighted complex network is one of such quantifying tools which are able to measure the microscopic differences of the chemical environments for a given node at varied scales by learning its *localized scaling dependence* from where it is located.

This underlying connection between the multi-fractal analysis and the microscopic view of the network complexity leads us to the straightforward implementation of our proposed localized scaling based network characterization approach. More precisely, we start with a given node *k* of the network and perform the SBw centered at it with *q* = 0 to learn the scaling dependence it experiences as we increase the scale *l* of the sandbox up to the network diameter *L*. This results in a localized scaling feature vector of tuples ${\mathcal{S}}_{k}={[{s}_{k,1}^{{\rm{{\rm T}}}},{s}_{k,2}^{{\rm{{\rm T}}}},\mathrm{...},{s}_{k,n}^{{\rm{{\rm T}}}}]}^{{\rm{{\rm T}}}}$, where *s*_*k*,*i*_ = (*logN*_*k*_(*B*(*l*_*i*_)), *log*(*l*_*i*_/*L*)). ${\mathcal{S}}_{k}$ is populated by the sampled logarithmic scaling dependence between the normalized box scale *l*/*L* and the number of nodes covered by the sandbox centered at *k*, hence *localized*. We repeat the process for every node of the network to construct a localized scaling feature space $\mathcal{S}(G)=\{{\mathcal{S}}_{k}|k\in {\mathcal{N}}]\}$ for the given network *G*. The localized scaling feature space $\mathcal{S}(G)$ is uniquely spanned by the localized scaling feature vectors of different network nodes. Its structure and properties are determined by the original network. Therefore, $\mathcal{S}(G)$ can be leveraged to characterize the network from a scaling dependence perspective. To the best of our knowledge, this is the first time that the localized scaling behavior of network is proposed as a quantitative profiling approach to characterize the structural characteristics of the complex network.

**Figure 18:**
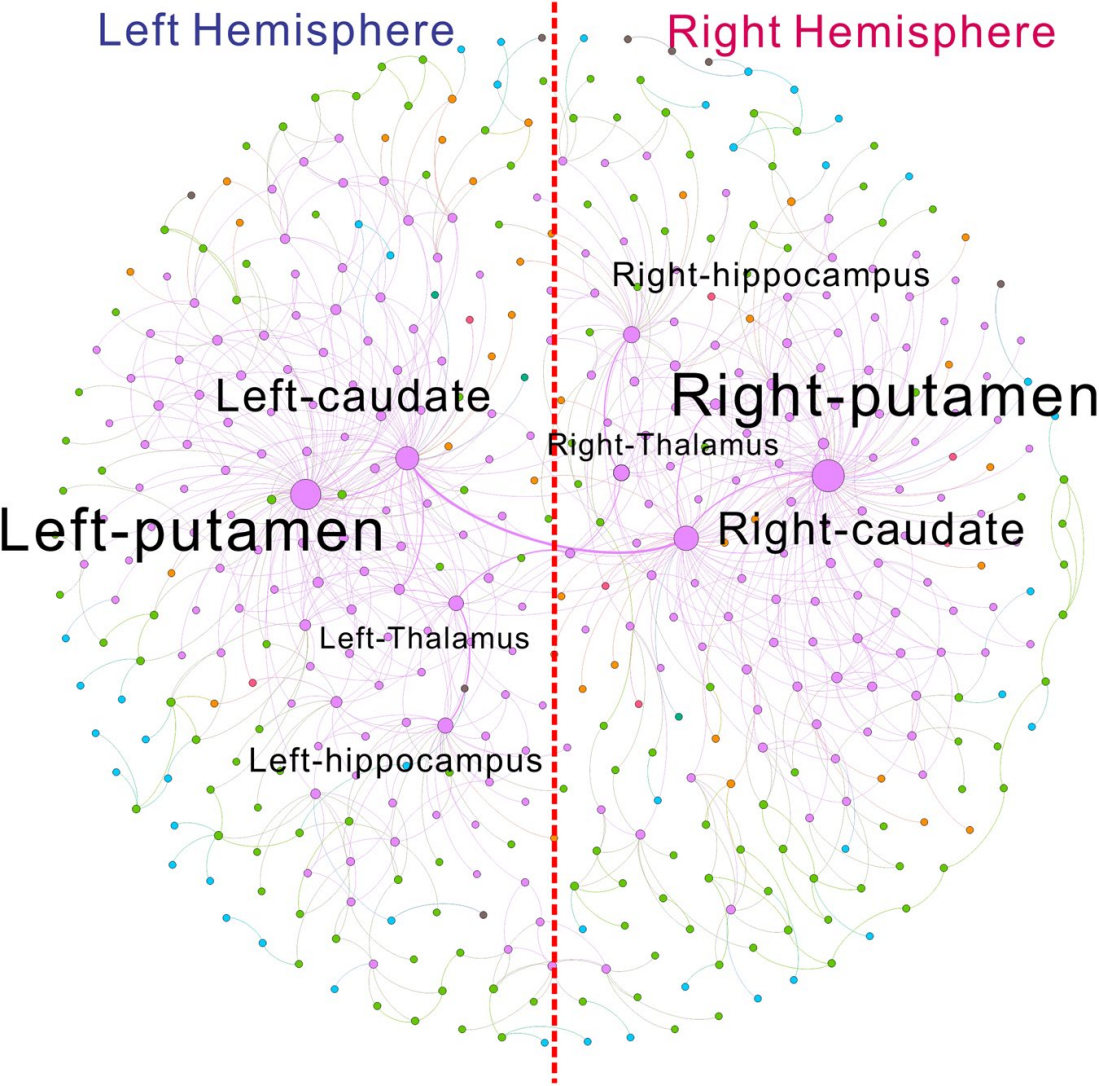
An example application of the proposed localized scaling feature space for characterization of weighted complex network. Interfaced with the unsupervised machine learning based clustering algorithm, the localized scaling based community detection is able to identify the brain network communities consistent with the anatomical facts. The detected communities are not limited to neighboring nodes but based on their relative spatial relations to the rest of network with potential functional implications.

An immediate application of this profiling approach is an easy integration with unsupervised machine learning algorithms to perform label-free detection of the network communities. The basic assumption is that the nodes sharing the same or similar scaling dependence localized to where they stand in the network should reside in similar chemical environments therefore belonging to the same network community. As a proof of concept, we performed the simple yet effective unsupervised *k*-means clustering algorithm with elbow method for network community detection on the Budapest human connectome and visualized the result in Fig. 18. This community detection approach identifies seven communities (colored differently in Fig. 18). The detection process is totally label-free with no prior knowledge of the functionality and locations of brain components and solely based on localized scaling feature vector of each node. Several key observations are due: i) brain network is symmetric so are the communities detected by the proposed approach, which is aligned with anatomical structure of human brain. ii) The dominant community (purple nodes) detected corresponds to the densely interconnected brain functional cluster formed by left and right Putamen, left and right Caudate, left and right Thalamus together with left and right Hippocampus. This community is consistent with the biological facts. The Putamen and Caudate are anatomically correlated to form the basal ganglia which is well known to be strongly interconnected with the cerebral cortex, thalamus, and brainstem to perform the control of voluntary motor movements and procedural learning. Thalamus is also manifoldly connected to the Hippocampus via the mammillo-thalamic tract and serves as an important relay station to propagate the sensory and motor signals to the cerebral cortex. This detected community serves as the major bridge between the two hemispheres and connectivity hubs to other functional entities thus sharing the similar chemical environment. iii) It can be noticed that the nodes belonging to the same community might not be necessarily immediate neighbors in contrast to the conventional modularity based communities. This is because the nodes are clustered based on their scaling dependence which is determined by the surrounding chemical environment at different scales. Therefore, it is possible that two nodes that are physically separated share the similar chemical environment to be labeled in the same community. For brain network, such chemical environment is a consequence of biological network evolution process and might have important functional implications that need to be explored in the future. In this sense, the concept of network community has been extended to characterize the node of the network from its relative spatial relation to the rest of the network. We hope the proposed localized scaling based network characterization and community detection can introduce a new research perspective for betterment of our understanding of the real world complex network in different domains.

## Discussion

The multi-fractal analysis has been long established to describe physical phenomena and objects by studying statistical scaling laws. The major attraction of its application stems from its capability to characterize the spatial and temporal irregularities that euclidean geometry fails to capture in real world physical systems, by an elegant interpretation of power-law behaviors. Its demonstrated effectiveness in characterizing complex systems motivates us to extend its formalism to the analysis of complex networks. However, the multi-fractality of weighted complex networks, the role of interaction intensity, influence of the underlying metric spaces and the design of reliable multi-fractality estimation algorithms are rarely discussed and remain an open challenge.

In this paper, we provide strong theoretical and experimental evidence for the intrinsic estimation bias of the previously proposed algorithms introduced by the incompatible growth of box scales and the implicit assumption that fractal scaling behaviors, if exist, hold at all scales of the networks. To overcome these disadvantages, we proposed two algorithms that can reliably estimate the multi-fractality of the network based on the critical points that correspond to a power-law scaling such that (i) it avoids box scales that lead to stagnant probability measures (i.e., staircase effect) and (ii) identifies the range of scales where a power-law scaling holds. In addition, we demonstrated that the estimation bias of the previously proposed algorithms deteriorates as a function of link weight distribution skewness and can not be compensated by either repeating the experiments or increasing the size of the networks (irrespective of the fact that it is practically difficult to scale the real world networks without changing its properties). More importantly, our work showed that the estimation can hardly be trusted if it assumes the existence of a scaling law that rules the network formation process throughout the scales. We provide real world weighted network examples where the observed distribution of scaling behaviors is localized in both space (e.g., co-existence of mono- and multi-fractal scaling dependency) and scales (e.g., power-law scaling valid over a finite range of scales).

Localized scaling behaviors reflect the fact that the network formation process of these networks is neither governed by a self-repeating iteration function system (IFS) that produces simple mono-fractal networks (e.g., Sierpinski fractal networks) nor a distribution of these IFSs throughout the scales of the network, which leads to a multi-fractal scaling behavior described by Eqs (13) and (17). Moreover, the formation of real world complex networks corresponding to different scales are constructed at different time points during the network formation process. The discontinuity of a power-law scaling at different scales therefore suggests that the network formation process is *dynamic* governed by *heterogeneous* forces as opposed to *stationary* models where either a fixed linking probability is assumed throughout the process (e.g., random graph theory) or a static linking policy (e.g., preferential attachment) is governing.

Furthermore, the network formation process and the resulting heterogeneous characteristics of real world networks also can not be fully explained from a pure structural and topological perspective. It is necessary to understand the role of the interaction intensity among the network components, the associated weight assignment process over time (e.g., how the weights change over time) and the metric space implicitly implied by the nature of these interactions (e.g., affinity relations, physical connectivities, causal dependences). We corroborate our argument with strong supporting evidence. More precisely, we identified both theoretically and experimentally two fundamental fractality transition phases that are governed by the intensity of network interactions (i.e., weights) and the embedded metric space defined on these networks:

(i) The scaling of the weights in a network formation process governed by an IFS determines the fractality of the network at a given scale. We reported the theoretical upper bound for the scaling factor *s* that transforms the network into a mono-fractal network given a) an IFS that leads to a family of Sierpinski fractal networks of unbounded size and b) the box size of an optimal covering method can shrink at same rate as the network grows. We argue that the observed multi-fractality of Sierpinski fractal networks by prior works does not necessarily come from a distribution of fractal scaling behaviors (i.e., multi-fractality) but can result from any deviation from these conditions (e.g., limited size and network growth rate). As a simple synthetic fractal network as the Sierpinski fractal network is, the weights and their distribution exhibit surprisingly powerful impact on the fractality of the networks. In a set of more realistic experiments, we further showed:
(ii) The weights and the metric space defined on real world networks arbitrate the existence of the fractality. By converting the collaboration network and the human brain connectome into binary networks, we decoupled the metric spaces defined on both networks from the link weights and transformed them to be a function of network topology alone. We demonstrated the removal of observed localized fractal scaling behaviors and an exponential scaling law (i.e., small-world property) takes place after the transformation of both networks. While keeping the topological configuration intact, the redefinition of metric space fundamentally altered the statistical scaling law of both networks.

This observation is not only important for the betterment of our understanding of the formation process of the real networks as the scaling behaviors reveal how the network grows. It is also a primary key to the network dynamics as the scaling behavior of the network plays a key role in governing the flows of the information across the network such as the rumor spreading in social networks or the protein exchange in a gene regulatory network. In these real world networks, the interaction intensity usually changes at a much more frequent pace compared to the changing rate of the network topology. For instance, a traffic network might stay structurally unchanged for a quite long time however the traffic volume (i.e., interaction intensity or weight) over its links varies constantly and fiercely. Given the fundamental role of link weights and metric space in determination of the scaling law, the time-varying network interactions can consequently impact the dynamics of the network. As a result, the failure to recognize the importance of link weight and metric space analysis will intrinsically limit our capability to characterize, predict and control the network behaviors.

Moreover, the variations of network scaling behaviors closely connect to the change of network properties, which leads us to solve a reverse research problem to characterize and quantify the heterogeneity of weighted complex networks by learning the scaling variations from a microscopic perspective of the network. We provide a general network characterization framework motivated by the observed locality and phase transition behaviors of the network scaling dependency. This characterization framework interprets the weighted complex network by the construction of a scaling feature space spanned by the localized scaling feature vectors determined both by the surrounding environment of individual nodes and the underlying metric space defined on the network. The proposed characterization is general and not limited to complex networks that are fractal or multi-fractal. It can be easily interfaced with subsequent analytical tools to unveil the intrinsic properties of the weighted complex network. As an important application, we showed the proposed characterization framework can be employed to learn the network communities that are consistent with our biological knowledge of the human brain connectivity patterns.

A very important aspect to emphasize is that the proposed characterization framework actually gives a general similarity metric within and between networks, which can be potentially leveraged as a basis for both structural and dynamic analysis on networks in a wide spectrum of applications. For instance, it can be interfaced with brain connectivity network constructed from real-time EEG measurements to identify tasks that correspond to different sets of scaling feature vectors, or to make both diagnosis and predictive analysis on brain-related pathological anomalies (e.g., traumatic damage, epilepsy) by learning corresponding scaling feature subspace. The proposed characterization framework can also be employed as self-similarity metric that enables the detection of anomalies or attack by comparing the learned scaling feature space to that during its normal operation mode in real time. In such cases, the benefit of the proposed characterization framework comes from its capability to quantify the variation of interaction intensities (e.g., change of transmitted power between grid node or maliciously injected traffic to overload the server) while no significant network structure mutation is present. On a different direction, this proposed framework also enables the fine-grain similarity analysis among a set of nodes in the same network. Aside from the presented network community detection based on this fine-grain similarity analysis, it is also useful to combine with domain knowledge (labels and attributes of nodes, e.g., functionality of brain region) to drive an informed exploration (e.g., any functional similarity between brain regions that are topologically apart but share the same scaling law). These examples may only constitute a small portion of its potential applications which necessitate our ongoing research efforts to extend the presented work to broader domains.

## Methods

### Multi-fractal analysis

Formally, let us consider a geometrical object tiled by boxes *B*(*l*) of size *l*. Let us define *L*, *M*_0_ and *M*_*i*_(*l*) as the linear length of the fractal, the total mass and the mass of the *i*-th box of size *l*, respectively. It is possible to determine *N*(*M*) that corresponds to the number of boxes sharing the same mass *M* given the object tiled by *B*(*l*). The probability density function of mass thus could be estimated by histograms in a double logarithmic plot *ln*(*N*(*M*)) against *ln*(*M*/*M*_0_) under different choice of box sizes *l*. The multi-fractal formalism^33^ states that if these histograms fall onto the same universal curve after rescaling both coordinates by a factor *ln*(*l*/*L*), the object is a geometrical multi-fractal^34^. Alternatively stated, the above property holds if

4 $$M \sim {M}_{0}{(l/L)}^{\alpha }$$

and

5 $$N(\alpha ) \sim {(l/L)}^{-f(\alpha )}$$

as the (*l*/*L*) → 0, where *α* is the Holder exponent which can be determined by,

6 $$\alpha =\frac{ln(\mu (B))}{ln(l/L)}=\frac{ln(M/{M}_{0})}{ln(l/L)}$$

*μ*(*B*) is an arbitrary measure defined on the support while it is equal to the probability to find a point in a given box. *N*(*α*) is the number of boxes with holder exponent *α*. *f*(*α*) is the *singularity* or *multi-fractal spectrum* if multi-fractal formalism holds^35, 36^. The multi-fractal spectrum shows the distribution of fractal dimensions across different sets of points sharing the same Holder exponent. Roughly speaking, it captures the variations in scaling behaviors of different subcomponents of the object. Equivalently, this variation could be captured by *generalized dimension D*(*q*),

7 $$\sum _{i}{M}_{i}{(l)}^{q} \sim {M}_{0}^{q}{(\frac{l}{L})}^{\tau (q)}$$

8 $$\tau (q)=(q-1)D(q)$$

as we take the limit *l*/*L* → 0. *τ*(*q*) is called as mass exponent. Distorting exponent *q* can be arbitrarily real-valued which serves to distinguish the irregularity in various regions of the object by magnification of measures scaled differently. The equivalence between the pair of (*f*(*α*),*α*) and (*τ*(*q*), *q*) is decided by the Legendre transformation,

9 $$\alpha =\frac{d\tau (q)}{dq}$$

10 $$f(\alpha )=q\frac{d\tau (q)}{dq}-\tau (q)$$

For a fractal object that can be characterized by a single fractal dimension, Eq. (7) suggests a sufficiently minimal fluctuation in measure *μ*(*B*_*i*_(*l*)) across all boxes of different sizes *l*. This directly translates to a narrowly distributed Holder exponent *α* and a *linear* dependence between mass exponent *τ*(*q*) and distorting exponent *q*. In contrast, a multi-fractal is rich in fluctuations of measure *μ* and have a spectrum *f*(*α*) widely spanned over *α* horizon and a *non-linear τ*(*q*) as a function of *q*. These fluctuations are to be captured and magnified via different choices of distorting factor *q*. To give some intuition, when *μ* is a probability measure as in box covering process, bigger weights in the summation of Eq. (7) will be placed to smaller probabilities if q is negative and to greater probabilities otherwise. The generalized fractal analysis approach is also well known as *multi-fractal analaysis (MFA)* that has wide applications due to its power to capture the heterogeneity underlying the structures of the objects.

### FBCw and FSBw

Box-covering and sandbox methods form the basis for MFA on weighed complex network with the following definitions.

*Box covering* method tiles the object of interest with boxes *B*(*l*) of different sizes *l*. An arbitrary measure *μ*(*B*_*i*_(*l*)) is defined for each box *B*_*i*_ that serves as support. Eq. (7) considers the case when *μ* is a probability measure such that,

11 $$\sum _{i}\mu {({B}_{i}(l))}^{q} \sim {(\frac{l}{L})}^{\tau (q)}$$

12 $$\mu {({B}_{i}(l))}^{q}={(\frac{{M}_{i}(l)}{{M}_{0}})}^{q}$$

when the limit *l* → 0 is considered. Therefore, the generalized fractal dimension calculated by box-covering method is given by,

13 $${D}_{bc}(q)=li{m}_{l\to 0}\frac{ln({\sum }_{i}{({M}_{i}(l)/{M}_{0})}^{q})}{ln(l/L)}\frac{1}{q-1}$$

Eq. (13) determines *D*_*bc*_(*q*) asymptotically from the scaling of number of non-empty boxes of decreasing size *l*.

*Sandbox* method investigates the scaling of an arbitrary measure *μ* within a region embedded in a metric space, i.e., a sandbox centered at certain point, as a function of its radius *l*. Formally, let $\mathcal{X}$ be the support of the measure *μ*. Let $\mathcal{D}:\mathcal{X}\times \mathcal{X}\to \mathcal{R}$ be a metric space defined on $\mathcal{X}$. For each ${x}_{i}\in \mathcal{X}$, we can define the following probability measure *M*_*i*_(*l*)/*M*_0_ as the chance to find an element ${x}_{k}\in \mathcal{X}$ with its distance to *x*_*i*_ in metric space $\mathcal{D}$ less than *l*,

14 $${\mu }_{i}(l)=\frac{{M}_{i}(l)}{{M}_{0}}=\frac{1}{{M}_{0}}\sum _{k\ne i}^{{M}_{0}} {\mathcal H} (l-\mathcal{D}({x}_{i},{x}_{k}))$$

Where ${M}_{o}=|\mathcal{X}|$ and ${\mathcal H}$ is the heaviside function. However, it is known that the relation ${\mu }_{i}(l) \sim {(l/L)}^{D}$, where *D* is the fractal dimension of the object, does not hold for all choices of sandbox centers as *l* → ∞ unless the center is the *origin* of the fractal^34^. Actually, sandbox method is equivalent to box covering method only if the choice of sandbox is *randomized*. We can rewrite Eq. (11) as,

15 $${(\frac{{M}_{i}(l)}{{M}_{0}})}^{q-1}\frac{{M}_{i}(l)}{{M}_{0}} \sim {(\frac{l}{L})}^{\tau (q)}$$

Equivalently,

16 $$E[{(\frac{{M}_{i}(l)}{{M}_{0}})}^{q-1}] \sim {(\frac{l}{L})}^{\tau (q)}$$

Therefore, the box-covering method can be understood as a sandbox method when the average is taken based on the measure distribution *M*_*i*_(*l*)/*M*_0_. Alternatively stated, the sandbox is equivalent to box counting only if choice of sandbox is randomized such that an estimate of *E*[(*M*_*i*_(*l*)/*M*_0_)^*q*−1^] can be obtained. Denote 〈.〉 as the operation to take average. We have,

17 $${D}_{sb}(q)=li{m}_{l\to 0}\frac{ln(\langle {({M}_{i}(l)/{M}_{0})}^{q-1}\rangle )}{ln(l/L)}\frac{1}{q-1}$$

We propose the finite box-covering method (FBCw) and the finite sandbox covering method for weighted networks (FSBw) to address the intrinsic estimation bias introduced by the incompatible growth rule of the box in numerical determination of multi-fractality of complex network with finite resolution. Formally, a complex network with a finite resolution is defined as follows,

**Definition 6 (finite resolution)**: *For a given weighted complex network G* = (*V*, *E*) *with distance metric* ${d}_{i,j}=\,\min \,\{{w}_{i,{k}_{1}}^{p}+{w}_{{k}_{1},{k}_{2}}^{p}+\cdots +{w}_{{k}_{n},j}^{p}\}$ *. The resolution of G is finite if and only if the shortest path distribution* ${F}_{{d}_{i,j}}(l)=P\{{d}_{i,j}\le l\}$ *has a discrete support set* ${\mathcal L} (G)=\{{l}_{k}|{F}_{{d}_{i,j}}({l}_{k})\ne {F}_{{d}_{i,j}}({l}_{k^{\prime} }),\forall k\ne k^{\prime} \}$.

The fundamental principle of FBCw and FSBw is to locate the scales of box that correspond to the compatible growth rule which is a function of the complex network *G*. For each node *v*_*i*_, the local compatible growth rule on *G* can be easily found by a strictly ordered set ${L}_{ < ,{v}_{i}}=\{{l}_{1},{l}_{2},\cdots ,{l}_{n},\}$ where *l*_*k*+1_ > *l*_*k*_ and ${L}_{ < ,{v}_{i}}\subseteq  {\mathcal L} (G)$. However, it is usually difficult to find a shared compatible growth rule across the network for the sandbox method or to analytically derive the optimal box-covering strategy as we did for the Sierpinski fractal network governed by a simple generation rule. In this context, we propose a data driven filtering method to interface with the box-covering and sandbox method for FBCw and FSBw. Both algorithms stand as a two-step process. In the first step, the accumulative measure ∑*μ*(*B*_*i*_(*l*))^*q*^ given the distorting factor *q* will be first obtained by growing the scale of the box *l* based on the unique path length of the network. Based on our discussion, this growth rule is generally incompatible. In the second step, we address this problem by a data-driven filtering procedure to obtain a subset *L*_<_ of the discrete support ${\mathcal L} (G)$ of ${F}_{{d}_{i,j}(l)}$ such that it is compatible with *G*. More precisely, FBCw and FSBw can be stated as follows:

**Step 1- Collecting the accumulative measure** ∑ *μ*(*B*_*i*_(*l*))^*q*^ **:**

- Given the distance metric *d*_*ij*_ on *G*, calculate all pairs of distances and encapsulate them into a matrix *D* either by the Floyd Warshall algorithm (*O*(|*V*|^3^)) or the Dijkstra algorithm (*O*(|*V*|(|*E*| + *Vlog*|*V*|))). Practically, if the graph is sparse in the sense that $|E|\ll {|V|}^{2}$, it is recommended to use Dijkstra algorithm which outperforms Floy-Warshall algorithm by a significant margin.
- Given the distance matrix *D*, derive the strictly ordered unique distance sequence *D*_<_  = {*d*_1_, *d*_2_, *d*_3_, …, *d*_*N*_} where *d*_*i*_ = *d*_*j*_ if and only if *i* = *j*. *d*_*N*_ is the diameter of the network. *D*_<_ serves as the tentative growth rule which, as discussed, is usually an incompatible growth rule that gives rise to the staircase effect whereas it can be alleviated by the subsequent filtering step. It should be also noted that the cardinality of *D*_<_ can be a computationally prohibitive in some cases when the number of unique path length is very large (e.g., |*D*_<_| of the collaboration network is close to 10^6^). In such cases, a resampling function $\mathcal{S}:{D}_{ < }\to {D}_{ < }^{{\prime} }$ where ${D}_{ < }^{{\prime} }\subseteq {D}_{ < }$ will be useful to bring down the computational overhead to an acceptable level. The proper choice of the resampling function is not constrained and may be subject to change based on the target network. In most of cases, a linear resampling function should be satisfactory.
- Iterate on *D*_<_ in an ascending order to perform the box-covering or sandbox covering procedure to obtain the accumulative measure *M*(*d*_*k*_) = ∑*μ*(*B*_*i*_(*d*_*k*_))^*q*^ at the scale *d*_*k*_ ∈ *D*_<_. No constraint is advised for the choice of a specific heuristic for this procedure. Practically, in the case that repeating the randomized box covering procedure for a large number of trials (to find the minimal number of boxes) is computationally impractical, Welsh–Powell algorithm usually gives satisfactory approximation after transforming the original network into its dual graph following the technique in ref. 28. Repeat the above procedure to obtain the accumulative measure sequence *M* = {*M*(*d*_1_), *M*(*d*_2_), …, *M*(*d*_*N*_)}.
**Step 2- Data-driven filtering for critical scales:** As a consequence of growing the scale of box based on an incompatible growth rule *D*_<_ for complex network *G* of finite resolution, there exists *d*_*i*_ and *d*_*i*′_ such that *M*(*d*_*i*_) = *M*(*d*_*i*′_) (i.e., the staircase effect). In practice, this condition can usually be relaxed to |*M*(*d*_*i*_) − *M*(*d*_*i*_′)| ≤ *ε* where *ε* is a tuning threshold and conditioned on the property of the network. Therefore, for every *d*_*i*_ ∈ *D*_<_, the major task of step 2 is to filter out all the *d*_*i*′_ where |*M*(*d*_*i*_) − *M*(*d*_*i*_′)| ≤ *ε* holds. Theoretically, it is possible to find a proper choice of *ε* such that the filtering can be done by enumeratively checking the condition for all choices of *d*_*i*_. However, picking the proper *ε* can be tedious manual process. In this context, we propose a simple yet effective variance based sliding window filter to identify the critical *d*_*i*_ that corresponds to a remarkable change in *M*(*d*_*i*_). Formally, the sliding window filter ${\mathcal F} ({\bf{x}},t)$
  18 $$ {\mathcal F} ({\bf{x}},t)=\sum ((\overline{x}-{x}_{i}{)}^{2})$$
  where *x* = [*x*_1_, *x*_2_, …, *x*_*W*_]^⊤^ is a *W*-dimensional vector of observations starting at *t*. *W* is the width of the sliding window. Then the data-driven filtering procedure can be stated as follows:
- Pick *d*_*i*_ from *D*_<_ in an ascending order and calculate ${\sigma }_{i}= {\mathcal F} (M,{d}_{i})$. Repeat it for all choices of *d*_*i*_ to obtain *σ* = {*σ*_1_, *σ*_2_, …, *σ*_*M*−*W*+1_}.
- Iterate on *σ* to find the index *i* of the peaks in *σ* that correspond to the critical scale *d*_*i*_.
- Perform the regression to Eqs (13) and (17) using the identified critical scales.

**Figure 19:**
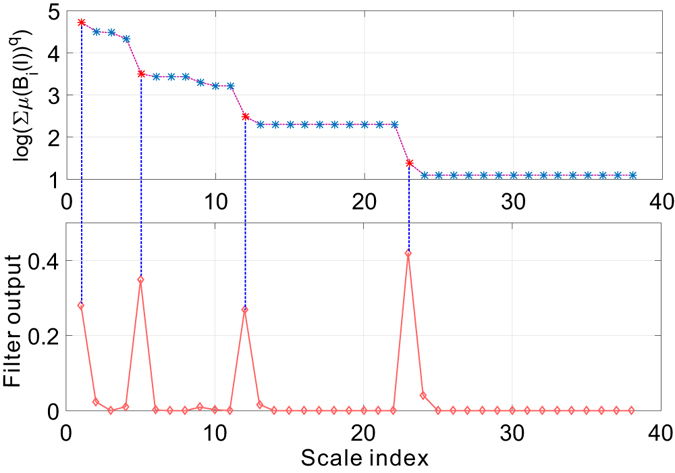
An example application of the proposed data-driven filtering method. By applying the filter sliding through the observations, the peaks of the output correspond to the critical scales where a significant change of the accumulative measure ∑*μ*(*B*_*i*_(*l*))^*q*^ occurs.

To better illustrate the efficacy of proposed data-driven filtering method, we plot in Fig. 19 the raw *M* against the scale index obtained in Step 1 by Welsh–Powell algorithm based box-covering strategy to Sierpinski fractal network *G*_4_ with *s* = 1/2 and *b* = 3. The peaks of *σ* exactly correspond to the critical scales *d*_*i*_ where a significant change of *M*(*d*_*i*_) appears. These scales are identified and used for numerical determination of the scaling behavior instead of all the scales to avoid stagnant observations.

Data accessibility: The datasets generated during and/or analyzed during the current study are available from the corresponding author on reasonable request.

## Electronic supplementary material

Supplementary Information
