# Supplementary material for: Reliable Multi-Fractal Characterization of Weighted Complex Networks: Algorithms and Implications
Source: Sci Rep. 2017 Aug 8;7:7487. doi: 10.1038/s41598-017-07209-5 (PMC5548933; doi:10.1038/s41598-017-07209-5)
Supplement: Supplementary file 1 — Supplementary Information [file 41598_2017_7209_MOESM1_ESM.pdf]

Supplementary Material for Reliable Multi-Fractal Characterization of Weighted Complex Networks: Algorithms and Implications

Yuankun Xue[1] **and Paul Bogdan**[1,*]

[1]Ming Hsieh Department of Electrical Engineering, University of Southern California, CA, USA, 90007
[*]Correspondence author, Email: pbogdan@usc.edu

ABSTRACT

Through an elegant geometrical interpretation, the multi-fractal analysis quantifies the spatial and temporal irregularities of the structural and dynamical formation of complex networks. Despite its effectiveness in unweighted networks, the multi-fractal geometry of weighted complex networks, the role of interaction intensity, the influence of the embedding metric spaces and the design of reliable estimation algorithms remain open challenges. To address these challenges, we present a set of reliable multi-fractal estimation algorithms for quantifying the structural complexity and heterogeneity of weighted complex networks. Our methodology uncovers that i) the weights of complex networks and their underlying metric spaces play a key role in dictating the existence of multi-fractal scaling and ii) the multi-fractal scaling can be localized in both space and scales. In addition, this multi-fractal characterization framework enables the construction of a scaling-based similarity metric and the identification of community structure of human brain connectome. The detected communities are accurately aligned with the biological brain connectivity patterns. This characterization framework has no constraint on the target network and can thus be leveraged as a basis for both structural and dynamic analysis of networks in a wide spectrum of applications.

1 Multi-fractal Analysis of Sierpinksi Fractal Network

Preliminaries: Before we present the analytical study of multi-fractality of Sierpinski network, we first formally introduce the definition of the Sierpinski family of our interest. For succinctness of statement, we introduce the following definitions:
◇ Let $b \in \mathscr{N}$ be a positively integer-valued number and $s \in (0,1]$ be a real number.
◇ Let $G = (V,E)$ be a network where V denotes the collection of nodes and E denote the collection of links.
◇ Let $D(v_i,G)$ denote the *degree* of v_i in network G and $W(e_{i,j})$ be the *weight* of link $e_{i,j}$
◇ A *tree* is an *undirected connected acyclic* network G.
◇ Given $G = (V,E)$ is a tree, let $\mathscr{L} : V \to V$ be a mapping function such that $D(v_i,G) = 1, \forall v_i \in \mathscr{L}(V) \subset V$. $\mathscr{L}(V)$ is also known as the set of *leaf* nodes of G.

Thus, we can formally introduce the definition as,

Definition 1 (Sierpinski network): *A family of Sierpinski networks is an infinite set of trees* $\mathscr{S} = \{G_0, G_1, ...G_n, ...\}$ *subject to the following constraints:*

(1) $G_0 = (V_0,E_0)$ where $|V_0| = 1$ and $E_0 = \emptyset$.

(2) For any $k \geq 1$, $\Delta G_k := G_k \setminus G_{k-1} = (\Delta V_k, \Delta E_k)$ where $\Delta V_k = V_k \setminus V_{k-1}$ and $\Delta E_k = E_k \setminus E_{k-1}$ such that the following conditions are all met:

(2.1) $D(v_i, \Delta G_k) = 1, \forall v_i \in \Delta V_k$

(2.2) $|\Delta V_k| = |\Delta E_k|$

(2.3) $\forall v_i \in \Delta V_k$, $\exists v_j \in \mathscr{L}(V_{k-1})$ such that $e_{i,j} \in \Delta E_k$

(2.4) For $k = 1$, $D(v_i, G_k) = b$ and for $k > 1$, $D(v_i, G_k) = b+1$, $\forall v_i \in \mathscr{L}(G_{k-1})$.

(2.5) For $k = 1$, $W(e_{i,j}) = W_0$, $\forall e_{i,j} \in \Delta E_k$. For $k > 1$, $W(e_{i,j}) = sW(e_{i',j'}), \forall e_{i,j} \in \Delta E_k$ and $\forall e_{i',j'} \in \Delta E_{k-1}$.

As Definition 1 states, the family of Sierpinski networks is constructed in an iterative fashion where the origin of the family is a single n_0 in G_0 and any member G_{k-1} is a subgraph of its successor G_k. By definition, a new family member G_k is introduced through the insertion of new nodes in ΔV_k and links in ΔE_k to its predecessor G_{k-1}. The construction of ΔV_k and ΔE_k is regulated by constraints (2.1)-(2.5). The constraints (2.1), (2.2) and (2.3) state each newly added node has one and only one link to the leaf nodes of G_{k-1}. Constraint (2.4) defines the growth rule of the Sierpinski family where each leaf node in G_{k-1} creates links to exactly b nodes in G_k. Constraint (2.5) decides the scaling factor of link weights between two generations. It is noted that we define the weights $W(e_{i,j})$ for an empty link set E_{-1} for maintaining the consistency of the

iterative definition.

To study the multi-fractality of Sierpinski network $\mathscr{S}$, we first need to map the network onto a metric space where the distance between a pair of nodes is defined as follows:

$$d_{i,j} = \min\{w^p_{i,k_1} + w^p_{k_1,k_2} + ... + w^p_{k_n,j}\} \tag{1}$$

where $w_{l,m} = W(e_{l,m})$ and link set $\{e_{i,k_1}, e_{k_1,k_2}, ..., e_{k_n,j}\}$ represents a path between v_i and v_j. The distance between a pair of nodes is thus decided by the path that minimizes the summation in Eq.(1). The exponent p is a constant decided based on the context of the network. Since p is fixed for a specific type of network considered, it is always possible to define $w'_{l,m} = w^p_{l,m}$. Thus we will assume $p = 1$ without loss of generality.

Lemma 1 (Diameter of G_k): *The diameter $D_{m,k}$ of $G_k \in \mathscr{S}$ is given by,*

$$D_{m,k} = 2W_0(\frac{1-s^k}{1-s}) \tag{2}$$

Proof: Due to the symmetry of G_k and constraint (2.5), the longest path length is twice the distance between $v_i \in \mathscr{L}(V_k)$ and n_0. Thus,

$$D_{m,k} = 2d(v_i, n_0) = 2(W_0 + sW_0 + s^2W_0 + ... + s^{k-1}W_0) = 2W_0\frac{1-s^k}{1-s} \tag{3}$$

□

Lemma 2 (Size of G_k): *The network size $|V_k|$ of $G_k \in \mathscr{S}$ is given by,*

$$|V_k| = \frac{1-b^{k+1}}{1-b} \tag{4}$$

Proof: According to the growth rule (2.4), $b * |\mathscr{L}(V_{k-1})|$ nodes will be newly introduced in the $k-$th iteration. As a consequence, the size of G_k can be calculated through the summation of a geometric progression,

$$|V_k| = 1 + b + b^2 + ... + b^k = \frac{1-b^{k+1}}{1-b} \tag{5}$$

□

To analytically investigate the multi-fractality of Sierpinkski family $\mathscr{S}$, we consider a box covering method $\mathscr{M}$ that tiles the $G_k \in \mathscr{S}, \forall k \in \mathscr{N}$ with boxes $\{B_i(l)\}$. For improving the clarity of the paper, we herewith formally introduce the following definitions:

Definition 2 (Box): *A box $B(l)$ is a subset of V such that for any pair of nodes v_i and $v_j \in B(l)$, $d_{i,j} \leq l$.*

Definition 3 (Compact box): *A box $B(l)$ is compact if and only if $d_{i,j} > l$ holds for $\forall v_i \in B(l)$ and $\forall v_j \in V \setminus B(l)$.*

Definition 4 (Box covering method): *A non-overlapping box covering strategy $\mathscr{M}$ is a partition of G such that $\cup B_i(l) = V$ and $B_i(l) \cap B_j(l) = \emptyset, \forall i \neq j$.*

Definition 5 (Optimal box covering): *A non-overlapping box covering strategy $\mathscr{M}$ is optimal if there does not exist $\mathscr{M}'$ such that $|\mathscr{M}'| < |\mathscr{M}|$.*

Theorem 1 (Optimal box covering): *A box covering strategy $\mathscr{M}$ is optimal if $B_i(l)$ is compact for $\forall B_i(l) \in \mathscr{M}$.*

Proof: Assume the optimal box covering is $\mathscr{M}'$ and $\mathscr{M}$ is compact. For arbitrary choice of a node v_j, $\exists B_i(l) \in \mathscr{M}$ such that $v_j \in B_i(l)$. Since $\mathscr{M}'$ is a partition of G, hence $\exists B_{i'}(l)$ such that $v_j \in B_{i'}(l) \in \mathscr{M}'$. Given $\mathscr{M}'$ is optimal, let us assume $B_{i'}(l) \setminus B_i(l) \neq \emptyset$ such that $\exists v_k \in B_{i'}(l)$ and $v_k \notin B_i(l)$. Equivalently, $\exists v_k \in V \setminus B_i(l)$ such that $d_{j,k} \leq l$. Consequently, $B_i(l)$ is not compact. This contradicts our assumption. Therefore, $B_i(l) = B_{i'}(l)$. Since our choice of v_j is arbitrary, we have $\mathscr{M} = \mathscr{M}'$.

□

It should be noted that a compact $\mathscr{M}$ is not a necessary condition for optimality of $\mathscr{M}$. Theorem 1 states that an optimal solution is compact as long as such a compact $\mathscr{M}$ exists. To give some intuition, let us consider a network with 4 nodes connected. Assume the link weight is uniformly set to 1 and the size of the box l to cover the network is 2. Obviously, the optimal covering strategy $\mathscr{M}$ requires 2 boxes. One covers the three nodes and the other covers the rest. No single box of size

2 can cover the entire network as the longest path is 3. Therefore $\mathscr{M}$ is trivially optimal. However, the boxes are not compact based on the Definition 3 as there always exists a node v_i to which the shortest path of $v_j \in G$ is smaller than l. Therefore, compactness is a stronger property of a covering method than its optimality.

Theoretically, the generalized fractal dimension via box-covering method is given by

$$D_{bc}(q) = lim_{l\to 0} \frac{ln(\sum_i (M_i(l)/M_0)^q)}{ln(l/L)} \frac{1}{q-1} \tag{6}$$

Eq.(6) holds only if the box covering strategy $\mathscr{M}$ is optimal. Interestingly, we are able to present a stronger box-covering strategy for Sierpinski family $\mathscr{S}$ and prove its compactness (hence optimality) conditioned on the value of s which is the scaling factor of link weights.

Optimal box covering for Sierpinski $\mathscr{S}$: The covering strategy starts with choosing how box size should be scaled in order to calculate the limit in Eq.(6) via a linear regression. In contrast to geometric fractal object that is well defined on Euclidean space, the complex network, once mapped to a metric space, has a *limited* resolution in a sense that we can not learn how the measure distribution $\sum_i((M_i(l))/M_0)^q$ changes over *continuously* scaled box sizes l. To address this problem for the considered Sierpinski family $\mathscr{S}$, we choose to grow the box size l by accumulating the *unique* link weights.

Formally, let us consider $G_k = (V_k, E_k) \in \mathscr{S}$ where $W(E_k)$ denotes the universal set of all its link weights. Let us define a strictly ordered set $\mathscr{W}_> = \{w_1, w_2, w_3, ..., w_n\}$ on G_k such that $w_i \in W(E_k), \forall w_i \in \mathscr{W}_>$ and $w_i < w_j$ if $i > j$. We define *box-size growth rule* as a set $\mathscr{L}$=$\{l_j | l_j = 2\sum_{i=j+1}^{n} w_i, j \leq n-1\}$. Once we set up the growth rule, the subsequent covering procedure can be stated as:

1. Given l_j, cover the subgraph in G_k rooted on a node $v_i \in \Delta V_j$ with a single box $B(l_j)$ of size l_j.
2. Repeat 1 for all the nodes in ΔV_j.
3. Cover every other node in G_k with a box of size l_j.
4. Steps 1-3 yield a box-covering strategy $\mathscr{M}_{opt}$.

Lemma 3 (Optimality of box covering): *$\mathscr{M}_{opt}$ is a compact optimal covering for $\mathscr{S}$ if $s < 1/3$.*

Proof: First, let us prove that a box of size l_j is able to cover the subgraph rooted on any node $v_i \in \Delta V_j$. Let us denote the diameter of the subgraph of G_k rooted on v_i as $D_{m,k-i}$. Given G_k is a tree such that the $D_{m,k-i}$ can be calculated as:

$$D_{m,k-i} = 2(s^{k-1}W_0 + ... + s^j W_0) = 2\frac{s^k - s^j}{s-1} W_0 \tag{7}$$

By definition, we know for $G_k \in \mathscr{S}$

$$l_j = 2\sum_{i=j+1}^{n} w_i \tag{8}$$

Where $w_i = s^{i-1}W_0$ and $n = k$. Thus $l_j = D_{m,k-i}$ such that the subgraph rooted on $v_i \in \Delta V_j$ can be covered with a single box.

Next, let us prove for any node $v_i \in \Delta V_p$ where $p < j$, it requires one and only one box $B(l_j)$ to cover it. We rewrite Eq.(8) as,

$$l_j = \frac{2s}{1-s} W_0 s^{j-1} - \frac{2s^k}{1-s} W_0 \tag{9}$$

The link weight between the node $v_i \in \Delta V_j$ and $v_{i'} \in \Delta V_{j-1}$ is equal to $w_j = W_0 s^{j-1}$. Given $s \in (0,1]$, the step 3 holds if $l_j < w_j$. A sufficient condition for that is

$$\frac{2s}{1-s} < 1 <=> s < 1/3 \tag{10}$$

Given that $w_j < w_{j-1} < ... < w_0 = W_0$, no two nodes v_i and $v_{i'} \in V_{j-1}$ can be covered by a box $B(l_j)$ if $s < 1/3$.

To finish our proof, we need to show that $\mathscr{M}_{opt}$ is compact. The proof follows the fact that for any box $B_m(l_j) \in \mathscr{M}_{opt}$, the minimal distance between a node $v_i \in B_m(l_j)$ and a node $v_{i'}$ outside the box $d_{i,i'}$ is no less than w_j while $w_j > l_j$. Thus, $B_m(l_j)$ is compact for any such box in $\mathscr{M}_{opt}$ so that $\mathscr{M}_{opt}$ is compact. By Theorem 1, the covering strategy $\mathscr{M}_{opt}$ is optimal.

□

Covering $\mathscr{S}$ by $\mathscr{M}_{opt}$ simply yields,

$$N(B(l_j)) = |\Delta V_j| + |V_{j-1}| = b^j + b^{j-1} + ... + b^0 = \frac{b^{j+1} - 1}{b-1} \tag{11}$$

We set up the basis on which we can analytically derive the multi-fractal spetrum. It requires to calculate the limit in Eq.(6). We consider mass distribution $M_i(l_j)/M_0$ as our probabilistic measure. It is noted that we have two types of equally sized boxes $B(l_j)$. Box type I covers every single node in V_{j-1} and the other covers the rest. The number of nodes they cover is given by,

$$M(l_j) = \begin{cases} \frac{b^{k-j+1}-1}{b-1}, & \text{Box type I} \\ 1, & \text{otherwise} \end{cases} \tag{12}$$

The probability measure $\mu(B_1)$ and $\mu(B_2)$ are calculated as,

$$\mu(B_1) = \frac{M(l_j)}{M_0} = \frac{b^{k-j+1}-1}{b^{k+1}-1} \tag{13}$$

$$\mu(B_2) = \frac{1}{M_0} = \frac{b-1}{b^{k+1}-1} \tag{14}$$

Assume $1 \ll j < k$,

$$\mu(B_1)^q = b^{-qj} \tag{15}$$

$$\mu(B_2)^q = b^{-qk} \tag{16}$$

Thus,

$$\sum \mu(B_i(l_j))^q = b^{(1-q)j} + b^{j-qk-1} \tag{17}$$

If we have $\lim_{j\to\infty}^{k\to\infty} j/k = O(1)$, which implies if we grow the Sierpinski network at the same speed as we shrink the box size l. We can simply write Eq.(17) as $b^{(1-q)j}$ given $j \to \infty$ such that the partition function $\tau(q)$ is obtained by,

$$\tau(q) = \lim_{j\to\infty} \frac{j(1-q)ln(b)}{(j-1)ln(s)+ln(A)} = (1-q)\frac{ln(b)}{ln(s)} \tag{18}$$

Eq.(18) suggests the partition function $\tau(q)$ is a *linear* function of q. In other words, the Sierpinski network $\mathscr{S}$ converges to a *perfect mono-fractal* if we were able to measure G_∞ given the scaling factor $s < 1/3$. In such case, the network could be characterized by a single fractal dimension $D = ln(b)/ln(s)$.

In practice, k is always finite. Let us assume that we are learning the scaling dependence from a scaling range $j_{min} < j < j_{max}$ and k is big enough to approximate G_∞ in the sense that $j \ll k$ and $k \gg 1$. If $q > 0$, we expect $\tau(q)$ stays as a linear function of q as this is same as the case discussed. However, $\tau(q)$ will be affected by the value of k when q is negative. This translates to the fact that we will observe $\tau(q)$ is a *non-linear* function of q that behaves different when it crosses the point $q = 0$.

Of particular note, such non-linearity of $\tau(q)$ has been reported in numerous prior works in which the studied Sierpinski network meets the mentioned conditions but is interpreted as *multi-fractality*. However, we argue that the observed non-linearity of $\tau(q)$ observed does not necessarily imply multi-fractality. The *limited size* of the graph considered with a *small* scaling factor could also be source of it, which does not mathematically link to multi-fractality.

2 Quantitative Analysis of Finite Resolution and Link Weight Distribution

***Estimation error analysis and stairway effect*:** To quantitatively perform error analysis in a more general case, let us denote $Y = \{y_1, y_2, y_3, ..., y_N\}$ and $X = \{x_1, x_2, x_3, ..., x_N\}$ as observation of output and input from a linear system. Let us assume the existence of a strictly linear dependency between Y and X and observations are perfect, i.e., error-free. Trivially, linear regression can give perfect estimate of the slope θ_0 *if only observations* in Y and X are considered. As the observations are *not* made continuously thus we can always insert fake observations as noise between any actual observations x_i and x_{i+1}. Let us introduce fake observations $Y_f = \{y'_{i_k} | y'_{i_k} = y_i, k \in [1,n]\}$ and $X_f = \{x_{i_k} | x_{i_k} = k * (x_{i+1} - x_i)/n + x_i, k \in [1,n]\}$. More precisely,

we are inserting fake observation pairs (Y_f, X_f) between x_i and x_{i+1} such that a small staircase is created because we assume all fake y observations of same value as y_i. Thus, the optimization problem can be written as :

$$\arg\min_{\theta^*} \sum_{k=1}^{i} (y_k - \theta^* x_k)^2 + \sum_{k=i_1}^{i_n} (y_i - \theta^* x_k)^2 + \sum_{k=i+1}^{N} (y_k - \theta^* x_k)^2 \tag{19}$$

For one dimensional linear regression, it can be shown that the slope of the fitted line θ is given by

$$\theta = \frac{\sum_i (x_i - \bar{x})(y_i - \bar{y})}{\sum_i (x_i - \bar{x})^2} \tag{20}$$

By introducing the fake observations, both $\bar{x}$ and $\bar{y}$ are affected and the new averages can be calculated as,

$$\bar{x'} = \frac{N\bar{x} + \frac{n}{2}(x_i + x_{i+1})}{N+n} \tag{21}$$

$$\bar{y'} = \frac{N\theta\bar{x} + n\theta x_i}{N+n} \tag{22}$$

To simplify our analysis, we assume $1 \ll n \ll N$ such that $\bar{x'} \approx \bar{x}$ and $\bar{y'} \approx \bar{y}$. Therefore, we can write Eq.(20) as,

$$\theta' = \theta \frac{\sum_{j=1}^{N} (x_j - \bar{x})^2 + \sum_{k=1}^{n} (x_i - \bar{x} + \delta k)(x_i - \bar{x})}{\sum_{j=1}^{N} (x_j - \bar{x})^2 + \sum_{k=1}^{n} (x_i - \bar{x} + \delta k)^2} \tag{23}$$

where $\delta = (x_{i+1} - x_i)/n$. Denote $\sigma_x^2 = \sum_{j=1}^{N} (x_j - \bar{x})^2$ and $\sigma_i = x_i - \bar{x}$. We have,

$$\theta' = \theta \frac{\sigma_x^2 + \sigma_i \sum_k (\sigma_i + \delta k)}{\sigma_x^2 + \sum_k (\sigma_i + \delta k)^2} = \theta \frac{\sigma_x^2 + n\sigma_i^2 + \delta\sigma_i n(n+1)/2}{\sigma_x^2 + n\sigma_i^2 + \delta\sigma_i n(n+1) + \delta^2 n(n+1)(2n+1)/6} \tag{24}$$

Given $n \gg 1$ and $\delta = (x_{i+1} - x_i/n)$, we define $W_s = x_{i+1} - x_i$ as the width of the "staircase" such that,

$$|\theta'| \approx |\theta \frac{\sigma_x^2 + n(\sigma_i^2 + \sigma_i \frac{1}{2} W_s)}{\sigma_x^2 + n[\sigma_i^2 + \sigma_i(\frac{1}{3}W_s^2 + W_s)]}| = F(\sigma_i, W_s, n)|\theta| \tag{25}$$

From Eq.(25), we can make the following observations:

i) The slope changing factor $F(\sigma_i, W_s, n)$ is a function of three factors: the deviation of x_i from the average of observations $\{x_i\}$, the width of the staircase and the number of fake observations inserted. The impact of the staircase on the estimation of the slope is decided by the location of the staircase, i.e., the sign of σ_i. When $\sigma_i > 0$ or the start of staircase x_i is greater than $\bar{x}$, $F(\sigma_i, W_s, n)$ is greater than 1 thus $|\theta'|$ is smaller than $|\theta|$. The introduction of fake observations, i.e., the staircase, will lead to underestimated (overestimated) slope of the fitted line given θ_0 is positive (negative). However, when $\sigma_i < 0$, the influence is reversed. Of particular note, the stair effect does affect the estimate if $\sigma_i = 0$.

ii) When $\sigma_i > 0$, the biased estimate decreases(increases if $\sigma_i < 0$) as the width of the staircase W_s grows. This is aligned with our intuition that if the value of fake observations $\{y_{i_k}\}$ get stuck at y_i over a very long x horizon, the fitted line tends to approach a line parallel with x-axis.

iii) Given fixed W_s and x_i, the number of fake observations n is proportional to deviation of θ from its actual value as the changing rate of the linear term in denominator of Eq.(25) is always greater than that in the numerator, rendering it a monotonically decreasing(increasing) function of n when $\sigma > 0$ ($\sigma < 0$).

To verify our theoretical findings, we use the same linear system $y = 50 - 0.4x$ and measure the estimation errors as a function of staircase location, number of fake observations and the width of staircase. Figure 1 shows the variation of the estimation error against different inserting locations of a staircase with number of fake observations ranging from 10 to 100 given a fixed $W_s = 100$. As predicted by our analysis, the sign of $(|\theta'| - |\theta_0|)/|\theta_0|$ changes across $\bar{x} = 100$ and its amplitude exhibits an almost asymmetrical pattern where the error shrinks to zero as x_i approaches $\bar{x}$ ($\sigma_i \to 0$) and becomes bigger otherwise. Given a fixed x_i, the increase of number of fake observations n will further bias the estimates. Figure 2 shows the influence of the width of the staircase which coincides with our analytical prediction.

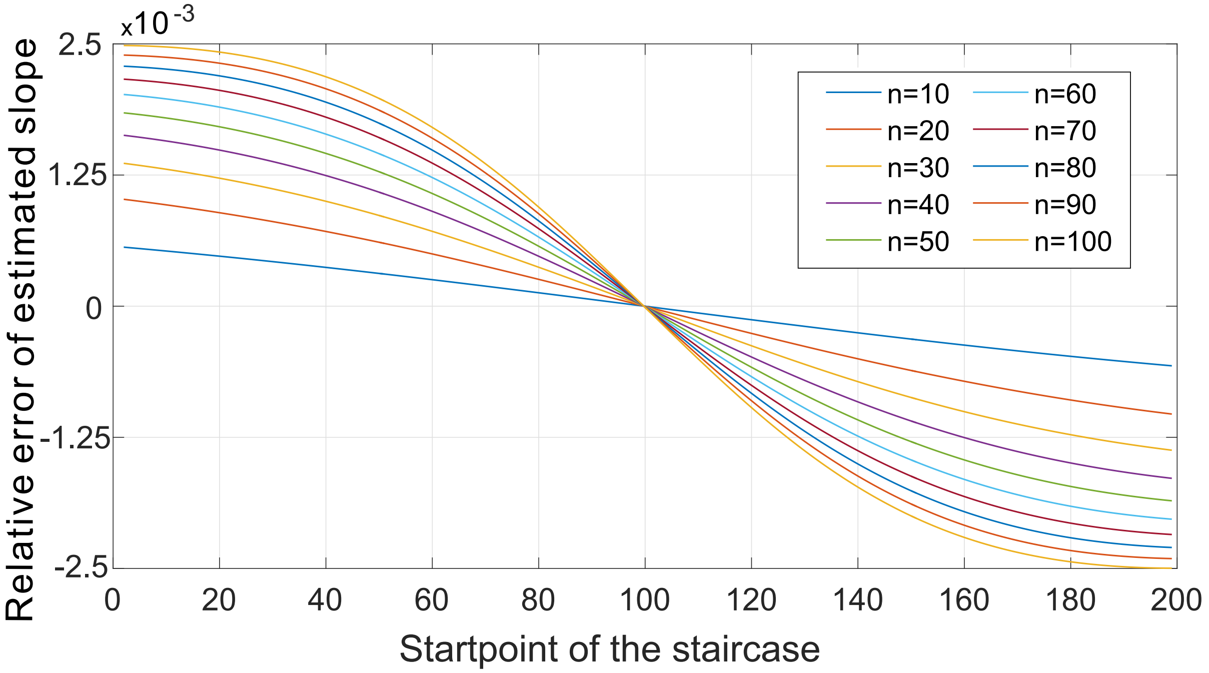

Figure 1. Relative error of estimate of θ_0 as function of insertion location of staircase and number of fake observations considered.

3 Phase Transition Characterized by Multi-fractal Free Energy Discontinuity

The transition point where the skewness of link distribution experiences a quick rising when scaling factor s decreases below a turning point suggests that a critical exponent can be potentially associated to this point to describe a transition from a mono-fractal phase to a multi-fractal phase (when considering networks of limited size) of Sierpinski fractal network. Based on the multi-fractal analysis of Sierpinski fractal network (see Lemma 3 in Supplementary Material Section 1), the Sierpinski fractal network converges to a perfect mono-fractal if the Sierpinski network grows at the same speed as we shrink the box size l. The growth rate of the Sierpinski network is fundamentally determined by the scaling factor s. Therefore, we expect that a proper choice of s in a given network G_i of *limited size* will serve as a phase transition point where Sierpinski network family *turns from a mono-fractal to a multi-fractal* as a result of violating our assumptions, i.e., identical growth rate as the box size in a graph of unbounded size.

In fact, we can analyze this transition from the perspective of thermodynamic free energy and investigate whether there exists a *discontinuity* in the derivatives of *free energy* function associated with the multi-fractal spectra of Sierpinski fractal networks [1]. Ehrenfest classification [3] labels the phase transition by the lowest derivative of the free energy that is discontinuous at the transition. For instance, the first order phase transition exhibits a discontinuity in the first derivative of the free energy with respect to some thermodynamic variable. In analogy to this thermodynamic concept, the *free energy of a multi-fractal* is defined as the mass exponent $\tau(q;s)$ that characterizes the scaling dependence of partition function $Z(l,q;s)$ on the size l of each partition (See [1,2] for detailed analysis).

$$Z(l,q;s) = \sum_i \mu(B_i(l);s)^q \sim (\frac{l}{L})^{\tau(q;s)} \tag{26}$$

We consider s in the partition function and mass exponent as they are determined by the scaling factor s if we fix the size of given Sierpinski fractal network. Similarly as thermodynamics classifies the phase transitions based on the behavior of the thermodynamic free energy as a function of other thermodynamic variables, we introduce a set of experiments to investigate the *first-order phase transition* of the free energy of Sierpinski fractal network as function of s. In the experimental setting, we choose G_4 from the Sierpinski fractal network family. G_4 has 364 nodes with a copy factor $b = 3$. We consider a series of decreasing scaling factor $s_i = 0.95^i$ where $i \geq 1$ being the index of the scaling factor. As a case study, we measured the $\tau(q;s_i)$ against different s_i with a fixed $q = -2$. We plot $\tau(q;s_i)$ against s_i to study the existence of discontinuity in Figure 3.

Figure 3.(a) suggests a sudden transition in the free energy of Sierpinski fractal network G_4 when we decrease scaling factor s below $s = 0.7738$. This can be further verified in the Figure 3.(b) where the first-order derivative of the free energy τ

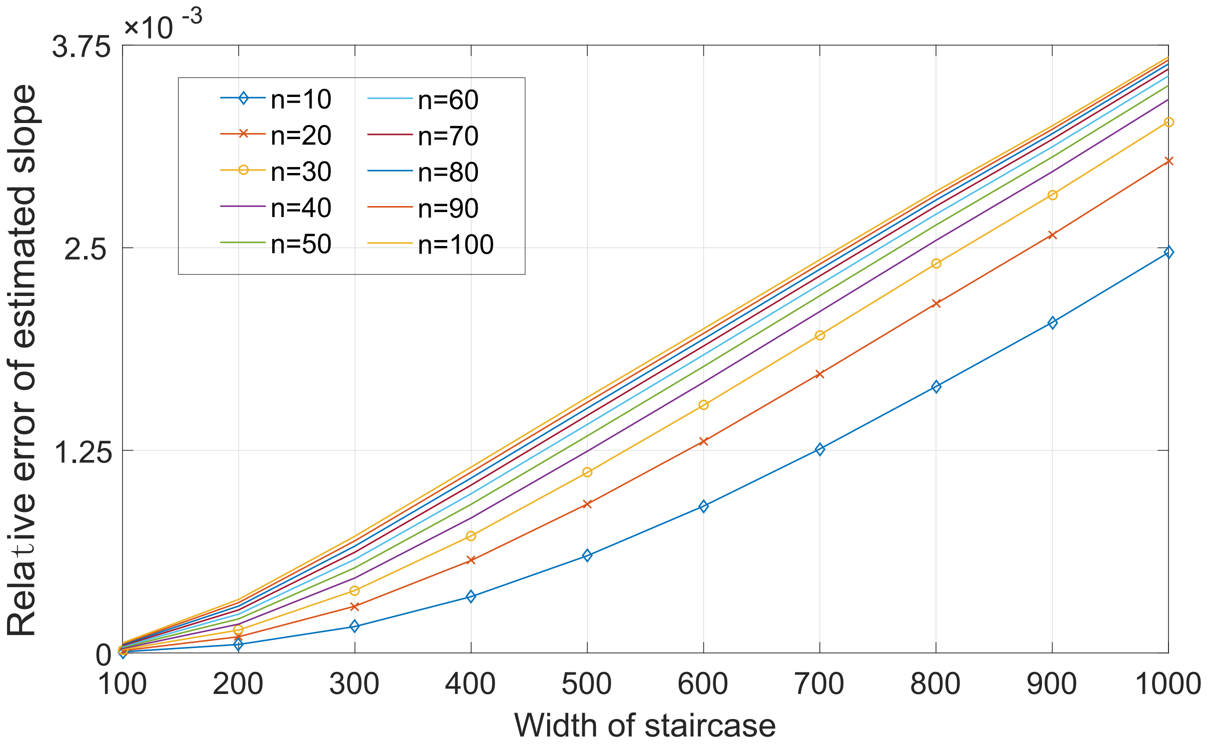

Figure 2. Relative error of estimate of θ_0 as function of width of staircase and the number of fake observations considered.

has a numerical singular point between $s = 0.7738$ and $s = 0.7351$. These two observations lead us to believe the existence of first-order discontinuity in the free energy function of Sierpinski G_4, i.e., the first-order phase transition. To further corroborate our claim that this phase transition suggests a change from mono-fractal phase to multi-fractal phase of Sierpinski fractal network, we investigate the dependence of free energy (mass exponent) $\tau(q)$ on distorting exponent q on the interested range of s between 0.9025 to 0.7351 and report it in Figure 4. By definition, the free energy (mass exponent) $\tau(q)$ of a mono-fractal exhibits linear dependence on q as opposed to a non-linear dependence on q for a multi-fractal. We can observe that the free energy $\tau(q)$ changes from a linear dependence behavior on q for s greater than 0.7351 to a nonlinear dependence as a function of q for $s = 0.7351$. This indicates G_4 stays a mono-fractal before this critical point and then transits to a multi-fractal beyond it. This observation coincides with Figure 3 and altogether they experimentally verify our claim that there exists a critical scaling factor s that leads to a phase transition. It should be also noted that this critical point might not precisely coincide with the transition point of skewness of link distribution and it is also influenced by the size of the network (e.g., the critical s for G_4 analyzed in the experiment might not coincide with that of G_5).

Reference

1. Lee, Jysoo, and H. Eugene Stanley. "Phase transition in the multifractal spectrum of diffusion-limited aggregation." Physical review letters 61.26 (1988): 2945.
2. Katzen, Dahn, and Itamar Procaccia. "Phase transitions in the thermodynamic formalism of multifractals." Nuclear Physics B-Proceedings Supplements 2 (1987): 517-520.
3. Jaeger, Gregg. "The Ehrenfest classification of phase transitions: introduction and evolution." Archive for history of exact sciences 53.1 (1998): 51-81.

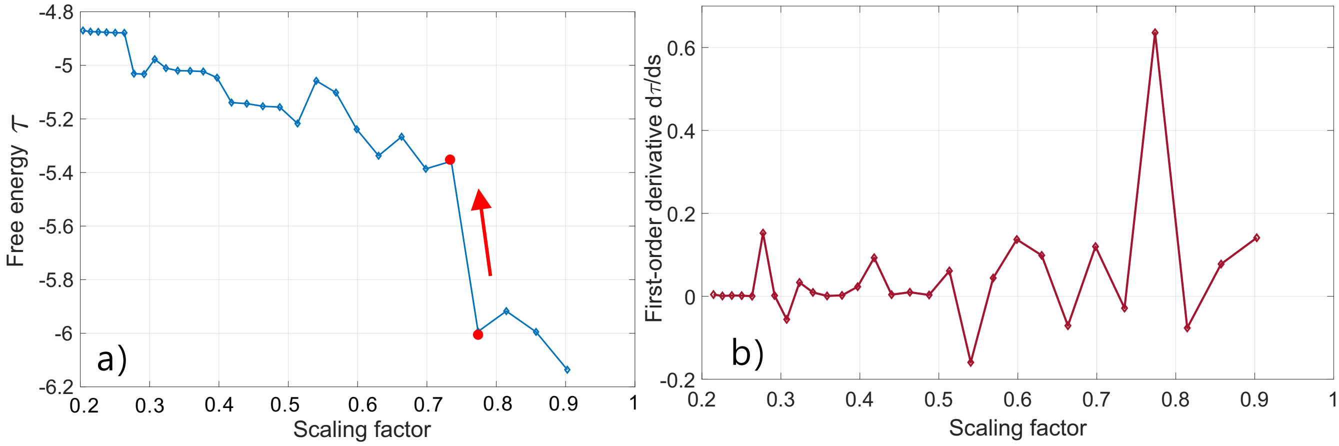

Figure 3. **First-order phase transition of the free energy τ as function of scaling factor s in Sierpinski fractal network G_4 with copy factor $b = 3$.** (a) The free energy of G_4 exhibits a discontinuous behavior between $s = 0.7738$ and $s = 0.7351$. (b) The observed possible discontinuity in the first-order derivative of free energy τ.

.

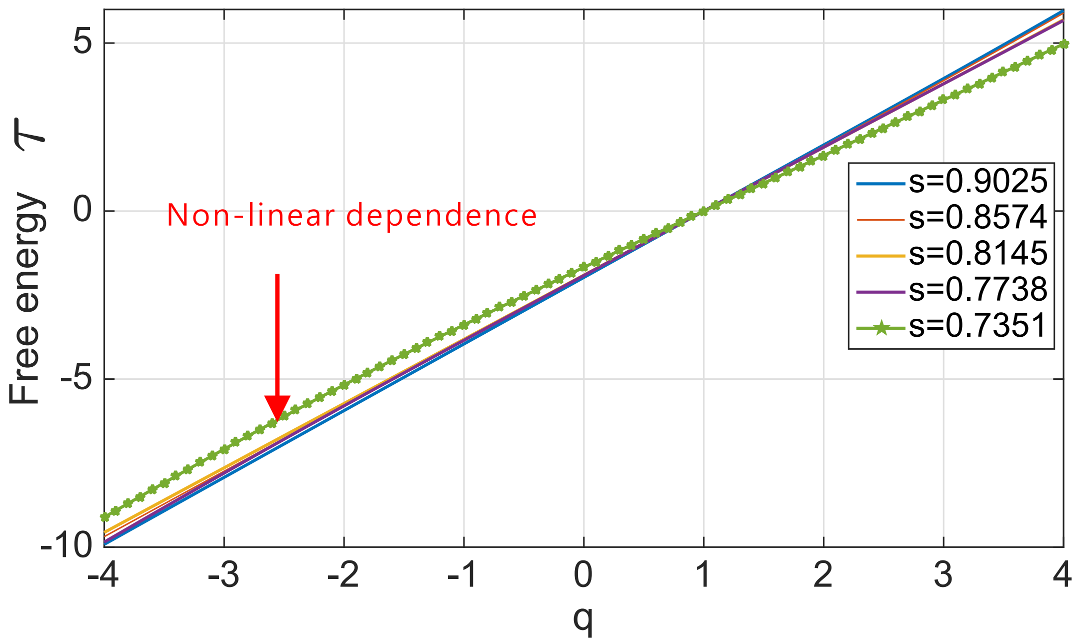

Figure 4. **Free energy (mass exponent) $\tau(q)$ deviates from linear dependence to non-linear dependence on q as scaling factor decreases below 0.7351.**

.
